# Reconfigurability‐Encoded Hierarchical Rectifiers for Versatile 3D Liquid Manipulation

**DOI:** 10.1002/advs.202405641

**Published:** 2024-07-29

**Authors:** Jiaqi Miao, Alan C. H. Tsang

**Affiliations:** ^1^ Department of Mechanical Engineering The University of Hong Kong Pokfulam Hong Kong 999077 China

**Keywords:** 3D liquid manipulation, interfacial flow, multifunctions, reconfigurable surfaces, soft material

## Abstract

Manipulating small‐volume liquids is crucial in natural processes and industrial applications. However, most liquid manipulation technologies involve complex energy inputs or non‐adjustable wetting gradient surfaces. Here, a simple and adjustable 3D liquid manipulation paradigm is reported to control liquid behaviors by coupling liquid–air–solid interfacial energy with programmable magnetic fields. This paradigm centers around a hierarchical rectifier with magnetized microratchets, using Laplace pressure asymmetry to enable multimodal directional steering of various surface tension liquids (23–72 mN m^−1^). The scale‐dependent effect in microratchet design shows its superiority in handling small‐volume liquids across three orders of magnitude (10^0^–10^3^ µL). Under programmed magnetic fields, the rectifier can reconfigure its morphology to harness interfacial energy to exhibit richer liquid behaviors without dynamic real‐time control. Reconfigured rectifiers show improved rectification performance in the inertia‐dominant fluid regime, i.e., a remarkable 2000‐fold increase in the critical Weber number for pure ethanol. Moreover, the rectifier's switchable reconfigurations offer flexible control over liquid transport directions and spatiotemporally controllable 3D liquid manipulation reminiscent of inchworm motions. This scalable liquid manipulation paradigm promotes versatile engineering and biochemistry applications, e.g., portable liquid purity testing (screening resolution <1 mN m^−1^), logical open‐channel microfluidics, and automated chemical reaction platforms.

## Graphical abstract

A new 3D liquid manipulation paradigm, coupling reconfigurable rectifiers and pre‐programmed static magnetic fields, enables selective directional transport (for 23–72 mN m^−1^ liquids) and spatiotemporally‐controlled biomimetic spreading. This active‐passive‐hybrid approach mitigates the trade‐off between simplicity and flexibility in liquid manipulation, showing potentials in portable liquid purity detection, logical open‐channel microfluidics, and automated biochemical platforms.

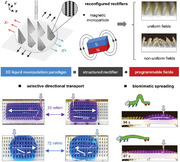

## 1 Introduction

Controllable liquid manipulation is essential for various applications, including microfluidics,^[^ ^1^, ^2^ ^]^ water harvesting,^[^ ^3^ ^]^ green energy,^[^ ^4^, ^5^ ^]^ thermal management,^[^ ^6^ ^]^ and biomedical applications.^[^ ^7^, ^8^ ^]^ Recent advancements have focused on passive and active liquid manipulation strategies to regulate liquid behaviors. A typical passive strategy is to exploit contact angle hysteresis to manipulate droplets on surfaces with chemical or topographic modulation of surface‐wetting gradients.^[^ ^9^, ^10^, ^11^, ^12^ ^]^ Other passive strategies explore bioinspired structured surfaces that capture important features of natural systems,^[^ ^13^, ^14^ ^]^ such as pitcher plant,^[^ ^15^ ^]^ spider silk,^[^ ^16^ ^]^ cacti,^[^ ^17^ ^]^ desert beetles,^[^ ^18^ ^]^ bird beaks,^[^ ^19^ ^]^ and Texas horned lizards.^[^ ^20^ ^]^ These bioinspired surfaces utilize interfacial energy to achieve continuous liquid control.^[^ ^21^, ^22^, ^23^, ^24^, ^25^ ^]^ However, these passive strategies are typically designed based on materials with fixed morphology, which may limit their adjustability in complex liquid transport problems with multi‐tasks. Alternatively, active strategies exploit external energies such as light,^[^ ^26^, ^27^ ^]^ thermal,^[^ ^28^, ^29^ ^]^ magnetic,^[^ ^30^, ^31^, ^32^ ^]^ ultrasonic,^[^ ^33^ ^]^ and electric fields^[^ ^34^, ^35^ ^]^ to control liquid dynamics. Namely, dynamic changes in external energy fields can alter surface morphology to transport liquids^[^ ^36^, ^37^, ^38^ ^]^ or create unbalanced forces to drive continuous liquid transport along arbitrary paths.^[^ ^39^, ^40^, ^41^, ^42^ ^]^ Although active strategies require dynamically varying external fields that may introduce extra complexity, they nonetheless afford greater flexibility in liquid control compared to passive approaches. The aforementioned active and passive strategies constitute the contemporary landscape of liquid manipulation technologies.

A persistent challenge in current liquid manipulation technologies is how to strike a balance between the inflexibility of passive strategies and the complexity of active approaches. Combining surface structures and material properties is a recognized promising strategy,^[^ ^43^, ^44^, ^45^ ^]^ motivating the development of passive‐active‐hybrid liquid manipulation techniques. A typical representative of hybrid methods involves regulating liquid interfacial dynamics via distinct static structure arrangements that are excited by constant external fields.^[^ ^46^, ^47^, ^48^, ^49^, ^50^, ^51^, ^52^, ^53^ ^]^ These hybrid methods improve the poor flexibility of passive structures and mitigate the complexity of active external field control in real‐time. Despite these advances, the monotonous liquid regulation mode of passive structured surfaces constrains achievable manipulation operations. Therefore, practical technologies that can enable richer liquid manipulation in a simpler manner are still being sought. The latest research indicates that fixed structured surfaces can enable diverse liquid behaviors.^[^ ^22^, ^23^, ^24^, ^54^ ^]^ Properly designing such surfaces and optimally coupling them with static external fields may provide a new avenue to address the core trade‐off and unlock satisfactory liquid manipulation technologies.

**Figure 1:**
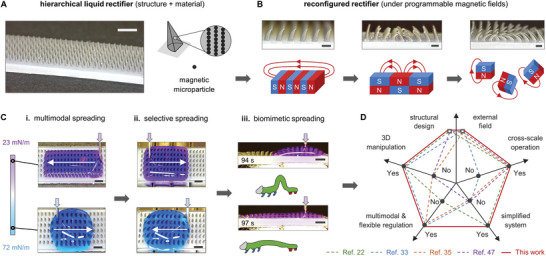
Our proposed 3D liquid manipulation paradigm. A) The hierarchically structured rectifier with magnetic‐responsive property. B) Reconfigured rectifiers under uniform, gradient, and other on‐demand programmed magnetic fields. C) i) Multimodal directional liquid spreading using original rectifier configuration. Reconfigured rectifiers enable ii) selective directional spreading; iii) biomimetic inchworm‐like spreading. D) Comparison of the proposed liquid manipulation technology with previous works.^[^ ^22^, ^33^, ^35^, ^47^ ^]^ Scale bars: 3 mm (A), 700 µm (B), and 2 mm (C,D).

Here, we propose an in‐depth design framework of reconfigurable structured rectifiers for multimodal 3D liquid manipulation with the aim of striking a balance between simplicity and adjustability. To this end, we develop systematic methodologies to integrate novel structured architectures and standardized static magnetic fields (**Figure** **1**A,B). The rectifier's hierarchical design supports multimodal directional liquid steering across a wide surface tension range (23–72 mN m^−1^) via unbalanced Laplace pressure or blocked liquid advancing contact line (Figure 1C–i), and enables precise cross‐scale liquid manipulation (10^0^–10^3^ µL). Instead of using dynamically varying external fields, our approach utilizes pre‐customized static magnetic fields generated by small‐volume NdFeB magnets to reconfigure the rectifier morphology into desired functional patterns. The simplified and controllable magnetic field eliminates the need for real‐time regulation and hence reduces complexity in manipulation. Our experimental results confirm that the uniform reconfiguration can achieve selective directional spreading of different surface tension liquids (Figure 1C–ii), which is achieved by arranging uniform magnetic fields with suitable strength and direction. Under non‐uniform magnetic fields, reconfigured rectifiers enable more complex morphologies and can create new 3D liquid manipulation modes that cannot be achieved by uniform magnetic fields, e.g., biomimetic inchworm‐like liquid spreading (Figure 1C–iii). Our liquid manipulation paradigm presents remarkable advances in key performance indicators compared to previous works (Figure 1D), and shows great potential in applications including liquid purity detection, multi‐channel logical liquid transport, and automated chemical reaction.

## 2 Results and Discussion

### 2.1 Fabrication and Characterization of The Rectifier

**Figure 2:**
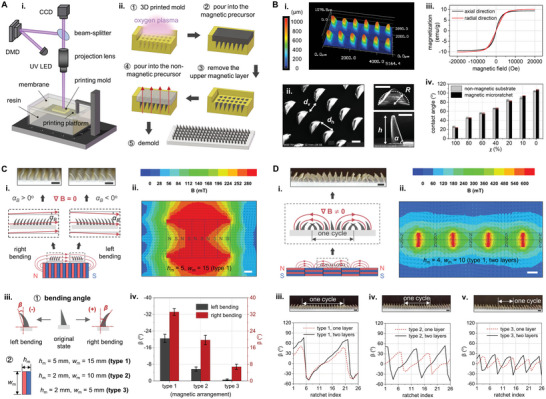
Fabrication, characterization and calibration of the proposed rectifier and magnetic fields. A) Illustration of the standardized rectifier fabrication, including i) the high‐resolution 3D printing platform description and ii) the magnetic‐filed‐assisted molding process. B) Characterization of the rectifier's i) surface topography, ii) detailed sizes, iii) magnetization properties, and iv) surface energy uniformity (mean ± SD). C) Construction of the standardized uniform magnetic fields: i) principle illustration and experimental diagram; ii) FEA simulation result (type‐1 magnets); iii) ② definition of microratchet bending angle *β* and ② the magnet types used to generate uniform magnetic fields; iv) calibration of the microratchet bending angle with respect to magnet type (mean ± SD). D) Construction of the standardized gradient magnetic fields: i) Illustration showing complex bending of microratchets; ii) simulation result (one layer of type‐1 magnets); iii–v) calibration of microratchet bending performance under different gradient magnetic fields, with experimental snapshots presenting one layer of three types of magnets. The ratchet index is named (from left to right) to show the bending angle variation of neighboring ratchets. Scale bars: 500 µm (B), 1 mm (i) of (C) and (i, iii–v) of (D), 3 mm (ii) of (C) and (ii) of (D).

We present a customized magnetic‐field‐assisted molding process to fabricate the rectifiers. The high‐resolution 3D printing technique, projection micro stereolithography, was employed to create uniform and precise microstructured molds (**Figure** **2**A–i; Figure S1, Supporting Information). The mold was first treated with oxygen plasma to eliminate impurities (Figure 2A–ii). A magnetic precursor composed of Ecoflex 00‐30 and iron microparticles was then filled into the mold as the rectifier's microratchets. After removing the upper layer magnetic precursor, the non‐magnetic Ecoflex 00‐30 precursor was added as the rectifier's substrate. Finally, a vertical magnetic field was used to align magnetic microparticles and impart the rectifier with an inherent “magnetization” direction (Experimental Section). After a curing period of 12 h, the rectifier can be obtained by demolding. Figure 2B–i shows the uniform 3D surface topography of a randomly selected region of the rectifier (with a 30×6 array of microratchets) observed under a 3D laser scanning microscope (Experimental Section). The actual microratchet dimensions closely match the design dimensions (Figure S2, Supporting Information), despite slight variations in height (mean ± SD: 973.8 ± 35.4 µm) due to molding errors. The SEM images (Experimental Section) in Figure 2B–ii provide definitions of the microratchet's size parameters, including height (*h* = 1200 µm), bottom radius (*R* = 500 µm), structural angle (*α* = tan ^−1^ (*h*/*R*) ≈ 67°), horizontal spacing (*d*_h_ = 900 µm), and vertical spacing (*d*_v_ = 600 µm). Further, we examine the magnetization properties of the rectifier by a vibrating sample magnetometer (Experimental Section). The data exhibits a narrow hysteresis loop with small coercivity and remanence, implying the characteristics of soft‐magnetic materials (Figure 2B–iii). In Figure 2B–iv, we assess the surface energy uniformity of the non‐magnetic substrate and magnetized microratchets. Similar apparent contact angles of experimental liquids on them indicate that the induced magnetic microparticles do not have a measurable influence on the wetting property of Ecoflex 00‐30. The experimental liquids are ethanol‐water binary mixtures with varied ethanol content (*χ*), where *χ* = *m*_e_/(*m*_e_ + *m*_w_), with *m*_e_ and *m*_w_ being the mass of ethanol and DI water. As *χ* decreases, the liquid's surface tension will conversely increase (Figure S3, Supporting Information) and present larger apparent contact angles (Figure 2B–iv).

### 2.2 Construction, Simulation, and Calibration of Standardized Magnetic Fields

High‐energy‐density NdFeB magnets are used to construct standardized uniform/gradient magnetic fields. We create uniform magnetic fields by horizontally aligning magnets of opposite poles (N and S), where attractive forces between magnets stick them together. This arrangement elongates the original gradient field of a single magnet and generates quasi‐uniform magnetic fields above and below the magnet array (Figure 2C–i). Ingeniously, the quasi‐uniform magnetic field has two parts with slight offset angles (*α*_*B*_) to the horizontal axis (left part: *α*_*B*_ >0; right part: *α*_*B*_ <0), allowing for selective actuation of right/left bending microratchets by utilizing their alignment with nearby magnetic field lines (Figure S4, Supporting Information). The magnetic field simulation validates these two parts in the quasi‐uniform magnetic field (Figure 2C–ii). We then calibrate the bending angle of microratchets *β* (left bending: *β* <0; right bending: *β* > 0) under various uniform magnetic fields (Figure 2C–iii), constructed by the magnets of different dimensions (width: *w*_m_ (mm); height: *h*_m_ (mm); length: *l*_m_ (mm)): type 1 (*w*_m_ = 15, *h*_m_ = 5, *l*_m_ = 30); type 2 (*w*_m_ = 10, *h*_m_ = 2, *l*_m_ = 20); type 3 (*w*_m_ = 5, *h*_m_ = 2, *l*_m_ = 15). Microratchet arrays display consistent values of *β* under uniform magnetic fields (small SD in Figure 2C–iv). Smaller magnet dimensions lead to reduced magnetic field strength, causing a corresponding decrease in *β*. To generate gradient magnetic fields, we employ the lateral attraction between magnets arranged in opposite orientations along their magnetization axis to combine them (Figure 2D–i). This magnet array arrangement actuates microratchets to present spatially varying bending angles periodically. The simulation result in Figure 2D–ii shows a gradient magnetic field distribution generated by a two‐layer arrangement of type‐2 magnets. We compare the gradient magnetic fields produced by three types of magnets in Figure 2 Diii–v. The microratchet arrangement period, given by the number of microratchets in one cycle, is decided by the magnet width *w*_m_. The range of *β* can be controlled by magnet dimensions and the number of stacked layers (Figure 2 Diii–v). Details on microratchet bending analysis, magnetic field construction, and simulation are in the Experimental Section and Figures S4–S8 (Supporting Information).

### 2.3 Multimodal Liquid Regulations via Hierarchical Structures

Our rectifier enables directional manipulation of liquids with different surface tensions, offering rich liquid transport directions. This is achieved through hierarchical designs incorporating the microratchet's bottom curvature and tilted body that can independently modulate various liquid directing modes.

#### 2.3.1 Mechanisms of Two Primary Liquid Directing Modes

**Figure 3:**
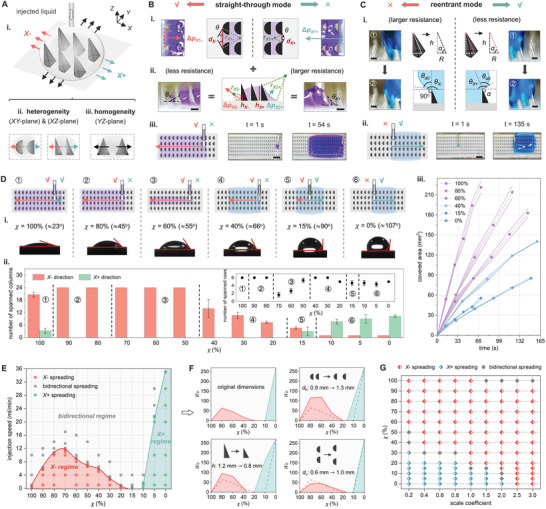
Mechanisms, multimodality, and generality of directional liquid spreading in the proposed rectifiers. A) Illustration of the i) liquid‐infused model, ii) heterogeneous, and iii) homogeneous cross sections of the microratchets. B) Straight‐through liquid spreading mode: mechanism illustration of Laplace pressure difference in the i) top view and ii) front view; iii) experimental illustration and snapshots. C) Reentrant liquid spreading mode: i) mechanism illustration of different dynamic advancing contact angles in the *X*− and *X*+ directions; ii) experimental illustration and snapshots. D) Six subdivided types of spreading modes under different liquid surface tensions: i) mode illustration and representative liquids (marked with apparent contact angle); ii) The liquid spreading columns/rows along *X*/*Y*‐axis of each mode. iii) Covered area changes of liquids with different surface tensions over time. E) Phase diagram of the injection flow rate and spreading modes. F) *We*‐*χ* phase diagrams of the rectifiers with changed size parameters. G) Phase diagram of the spreading modes for the rectifiers at different scales. Scale bars: 500 µm (i, ii) of (B) and (i) of (C), 3 mm (iii) of (B) and (ii) of (C).

Our analysis is based on a liquid‐infused model (**Figure** **3**A–i; Figures S9 and S10, Supporting Information), which considers thirty columns in the *X*‐axis and six rows in the *Y*‐axis. We set the injection flow rate at a low speed of 130 µL min^−1^ to neglect the inertia effect. The liquids were dyed in purple (χ≥50%) and blue (χ<50%) to improve visualization (Experimental Section and Figure S3, Supporting Information). Microratchets feature a transition cone connecting the bottom semicircular structure to the top structure, with heterogeneous cross sections in the *XY*‐/*XZ*‐plane and a homogeneous cross section in the *YZ*‐plane (Figure 3A–ii, iii). The primary axis for directional liquid spreading is the *X*‐axis, while the *Y*‐axis spreading is symmetric. Surface tension variations result in different liquid accumulation heights, measured by the distance between the liquid's top interface and the rectifier substrate's top surface along the *Z*‐axis.

The rectifier employs hierarchical structures to direct various surface tension liquids, categorized into straight‐through and reentrant modes (Movie S1, Supporting Information). The straight‐through mode utilizes Laplace pressure asymmetry at the liquid‐air interface, facilitated by bottom curvature and inclined sides of microratchets, to direct low‐surface‐tension liquids toward the *X*− direction (Figure 3B). Considering the liquid front curvature in top view (1/*r*_1_, Figure 3B–i) and front view (1/*r*_2_, Figure 3B–ii), the Laplace pressure in the spreading direction (Δ*p*_*X*__‐_) and the pinning direction (Δ*p*_*X*__+_) is expressed as:

(1)

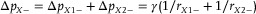



(2)

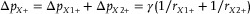

where *γ* denotes the liquid surface tension. Δ*p*_*X*__1+_ and Δ*p*_*X*__1‐_ are the Laplace pressure components due to the bottom curvature of microratchets in the spreading direction and the pinning direction, respectively. Δ*p*_*X*__2+_ and Δ*p*_*X*__2‐_ are the Laplace pressure components due to inclined sides of microratchets in the spreading direction and the pinning direction, respectively.

In the top view, the distinct convex liquid‐air interfaces result in different resistances on two sides (Figure 3B–i). Using trigonometry, the Laplace pressure becomes:

(3)

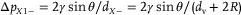



(4)

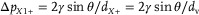

where *d*_*X*__‐_ and *d*_*X*__+_ are the length of liquid propulsion lines in the *X*− and *X*+ directions. In the front view, the concave liquid–air interface promotes liquid spreading (Figure 3B–ii) via Laplace pressure given by:

(5)

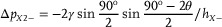



(6)

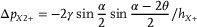

where *h*_*X*__−_ and *h*_*X*__+_ (*h*_*X*__−_ < *h*_*X*__+_) represent the liquid height in the spreading and pinning directions. *α* is the structural angle of the microratchet (*α* < 90°). When substituting Equations (3–6) into Equations (1, 2), Δ*p*_*X*__−_ and Δ*p*_*X*__+_ are finally described as:

(7)

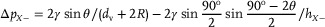



(8)

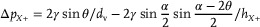




Since |Δ*p*_*X*__1‐_| < |Δ*p*_*X*__1+_| and |Δ*p*_*X*__2‐_| > |Δ*p*_*X*__2+_|, we have Δ*p*_*X*__−_ − Δ*p*_*X*__+_ < 0, meaning the less total resistance in the *X*− direction than that in the *X*+ direction (Figure S11A, B, Supporting Information). The resistance difference is evident in a smaller dynamic advancing contact angle in the *X*− direction (Figure 3B–ii, *θ*_*X*__−_ < *θ*_*X*__+_, where *θ*_*X*__−_ and *θ*_*X*__+_ are the dynamic advancing contact angles in the *X*− and *X*+ directions), and eventually drives the directional liquid transport. Figure 3B–iii presents an example of the directional transport of the low surface tension liquid with χ=90% (Movie S1, Supporting Information). Within ≈54 s, the liquid spans a distance of ≈14.4 mm toward the *X*‐ direction.

Increasing liquid surface tension reduces wettability and causes a switch from the straight‐through mode to the reverse reentrant mode (Figure 3C; Movie S1, Supporting Information). The injected liquid initially repels the microratchet structure but eventually enters the microratchet gaps due to accumulated gravity, representing a transition from the Cassie–Baxter state^[^ ^55^ ^]^ to the Wenzel state.^[^ ^56^ ^]^ The strong pinning effect from Laplace pressure leads to accumulated liquid height surpassing the microratchet height. The liquid spreading direction is hence determined by the heterogeneous top structure of microratchets, unlike the straight‐through mode determined by the bottom curvatures and the inclined sides. As shown in Figure 3C–i, asymmetric reentrancy effectively blocks the advancing contact line in the *X*‐ direction. The actual advancing contact angles of the liquid along the *X*− and *X*+ directions (*θ*_*X*__−_ and *θ*_*X*__+_) are:

(9)

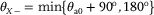



(10)

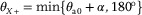

where *θ*_a0_ is the liquid's intrinsic advancing angle. Since *α* <90°, *θ*_*X*__+_ is smaller than *θ*_*X*__−_ when *θ*_a0_ + *α* < 180°, indicating a lower forward resistance in the *X*+ direction (Figure S11C, Supporting Information). Thus, the reentrant liquid spreading is directed toward the *X*+ direction (state ②–②, Figure 3C–i), which enables continuous directional transport of pure DI water across ≈10.8 mm within ≈135 s (Figure 3C–ii).

#### 2.3.2 Multimodal Liquid Spreading Behaviors

Evolved from the two primary directing modes, liquid spreading in 3D (*XY*‐/*YZ*‐/*XZ*‐plane) varies with different surface tensions (*χ* = 100%–0%), which can be subdivided into six modes (Figure 3D–i: Figure S12 and Movie S1, Supporting Information). In mode ②, the liquid follows the straight‐through mode, while exists slight spreading to the *X*+ direction due to extremely low surface tension (Δ*p*_*X*__1+_ ≈ 0). When the liquid surface tension increases slightly, this phenomenon will be eliminated and pure straight‐through mode occurs. With decreased *χ*, the liquid sequentially undergoes: spreading across all six rows in the *Y*‐axis under small Laplace pressure resistance (mode ②); dropping in spanned rows in the *Y*‐axis due to enhanced Laplace pressure resistance (mode ③); spreading across all six rows again in the *Y*‐axis because the liquid begins to climb over microratchets (mode ④). Further decrease in *χ* results in a transition from straight‐through to reentrant mode (mode ⑤), exhibiting bidirectional spreading. Finally, pure reentrant mode occurs for high surface tension liquids (mode ⑥). Figure 3D–ii shows the number of spanned microratchet columns/rows to illustrate the mode transition. The differences are manifested in the variation of spreading in the *X*− and *X*+ directions, as well as in whether the liquid can span all six rows in the *Y*‐axis. The transition between the two primary modes occurs at ≈*χ* = 15%. When χ≤40%, the liquid falls from the rectifier along the *Y*‐axis, therefore showing a decreased number of spanned columns along the *X*‐axis. Moreover, higher surface tension liquids repel the rectifier and have a larger accumulation height, hence resulting in a slower increase in the spreading area in the *XY*‐plane over time (Figure 3D–iii). The related liquid spreading velocity for each case is examined in Figure S13 (Supporting Information).

#### 2.3.3 Generality Evaluation of The Rectifiers

We now proceed to evaluate the generality of our rectifier design to understand its performance under different application scenarios, including high flow rates where inertia affects directional transport, as well as different rectifier scales. Figure 3E presents a phase diagram depicting the influence of injection flow rates on liquid spreading modes. The critical flow rate in the straight‐through mode initially increases and then decreases with increasing liquid surface tensions (*X*− regime). For the reentrant mode, the critical flow rate continues to rise with increasing liquid surface tension. We use the dimensionless Weber number (*We*) to quantitatively evaluate the inertial effect versus surface tension on directional liquid spreading:

(11) $$\mathrm{We}=16\rho v^{2}/\left(\pi^{2}D^{3}\gamma \right)$$

where *ρ*, *v*, and *D* are the liquid density, injection flow rate, and the inner diameter of the syringe needle, respectively. By solely modifying *h* (1.2 mm → 0.8 mm), *d*_h_ (0.9 mm → 1.3 mm), or *d*_v_ (0.6 mm → 1.0 mm), we obtain different *We*‐*χ* phase diagrams (Figure 3F and Experimental Section). The overall trend of critical flow rates remains consistent when slightly changing dimensions (<±50%). Reducing *h* facilitates liquid crossing over the microratchet and leads to an earlier transition between two primary modes. Widening *d*_h_ allows for more space for liquid accommodation between microratchets, leading to a higher critical *We* peak for the straight‐through mode. Conversely, widening *d*_v_ elongates the liquid propulsion line, thereby reducing the critical *We* (Figure S14, Supporting Information).

We then investigate how microratchet arrays of different scales may influence their performance in liquid manipulation. We define a scale coefficient to represent scaling down (<1) or scaling enlargement (>1) with respect to the original ratchets (Figure S15, Supporting Information). Figure 3G shows the regulation mode variation of ratchets with different scale coefficients. Reducing the ratchet scale preserves multimodal liquid manipulation, but large‐scale ratchets keep a single straight‐through mode for all liquids (Figure S16, Supporting Information). This scale‐dependent effect of our ratchet structures emphasizes their suitability in precise small‐volume liquid manipulation, typically in 10^0^–10^3^ µL.

### 2.4 Reconfigured Rectifiers with Enhanced Flexibility in 3D Liquid Manipulation

By utilizing the rectifier's magnetic‐responsive property, we develop a design‐on‐demand platform that enables both multimodality and adjustability in liquid manipulation. We apply static external magnetic fields to reconfigure the rectifier's structural features to adjust liquid manipulation. This method allows us to couple external fields and the liquid–solid–air interfacial energy system, distinguishing itself from current liquid manipulation technologies.^[^ ^13^, ^43^, ^44^, ^57^, ^58^ ^]^

**Figure 4:**
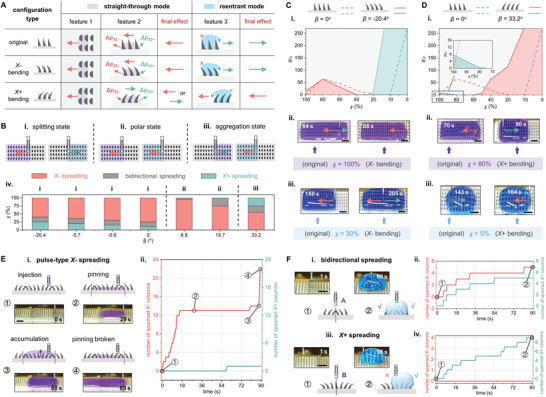
Enhanced flexibility of reconfigured rectifiers in 3D liquid manipulation. A) The core strategies about how different rectifier configurations (*X*−/*X*+ bending) can adjust liquid behaviors. B) Illustration of three states of reconfigured rectifiers: i) splitting state; ii) polar state; iii) aggregation state; iv) the completion of their conversion under different magnetic field strengths and directions. C) Improved rectification performance of the *X*− bending rectifier: i) adjusted *We*‐*χ* phase diagram; ii) enhanced rectification effect on pure ethanol; iii) earlier transition in spreading mode for the χ=30% liquid. D) Improved adjustability of the *X*+ bending rectifier: i) reversed *We*‐*χ* phase diagram; ii) the χ=80% liquid with changed spreading direction from *X*− to *X*+ direction; iii) the χ=5% liquid with changed spreading direction from *X*+ to *X*− direction. E) Under the gradient magnetic field, the straight‐through mode of the rectifier develops into the pulse‐type *X*− spreading: i) illustration of its four main stages; ii) the spanned column number in *X*− and *X*+ directions over time. F) Liquid spreading in reentrant mode is affected by the injection position: i, ii) in position A, the liquid follows bidirectional spreading. iii, iv) in position B, the liquid follows *X*+ directional spreading. Scale bars: 3 mm (C–F).

**Figure** **4**A illustrates the relationship between configuration types, microratchet's structural features, and spreading modes. *X*− and *X*+ bending configurations maintain the bottom curvature (feature 1), but alter the microratchet's tilting angle (feature 2), and top heterogeneity (feature 3). Specifically, the *X*− bending configuration improves the rectification performance by increasing structural asymmetry. For the straight‐through mode, the increased difference between Δ*p*_*X*__2−_ and Δ*p*_*X*__2+_ leads to a larger pinning effect in the *X*+ direction. For the reentrant mode, the enhanced top heterogeneity reduces *θ*_*X*__+_ and enlarges *θ*_*X*__−_ (Figure S17, Supporting Information), keeping the original contact angle hysteresis. In contrast, the *X*+ bending configuration changes the original liquid manipulation rules completely. Δ*p*_*X*__2‐_ and Δ*p*_*X*__2+_ guide the liquid to spread toward the *X*+ direction, countering the effect of the bottom curvature and causing uncertainty in the transport direction of the straight‐through mode. In addition, the *X*+ bending microratchets reverse the initial top heterogeneity and make the reentrant mode change from its original *X*+ direction to the *X*− direction (Figure S17, Supporting Information).

The liquid behaviors guided by uniformly reconfigured rectifiers can be categorized into three states: splitting, polar, and aggregation (Figure 4 Bi–iii). The splitting state, observed in the original and *X*− bending configurations, directs low and high surface tension liquids toward the *X*− and *X*+ directions, respectively. The polar state, with a slight *X*+ bending, retains the spreading direction in the straight‐through mode, but the adjusted top heterogeneity switches the reentrant direction. The aggregation state, which substantially increases the *X*+ bending, reverses the direction of both two primary modes. By applying different uniform magnetic fields to change *β* (Figure 2C–iv), we obtain the dominance of these three states for different surface tension liquids (Figure 4B–iv). We illustrate the changes in liquid transport behaviors by considering the updated *We*‐*χ* phase diagrams for the *X*− bending configuration (mean: *β* = −20.4°) and the *X*+ bending configuration (mean: *β* = 33.2°) (Figure 4C–i,D–i; Movie S2, Supporting Information). The *X*− bending configuration transforms the ethanol spreading from slightly bidirectional to strictly directional (Figure 4C–ii) and increases the critical *We* by 2000‐fold (from 0.0076 to 15.775). It also shifts the original transition region between two primary modes and broadens the operable liquid surface tension range of the reentrant mode. For instance, the liquid with χ=30% transitions from *X*− spreading to bidirectional spreading (Figure 4C–iii). In contrast, the *X*+ bending rectifier reverses the original directing directions. The χ=80% liquid shifts from *X*− to *X*+ spreading (Figure 4D–ii), while the χ=5% liquid shifts from *X*+ to *X*− spreading (Figure 4D–iii). These results demonstrate the flexibility of uniformly reconfigured rectifiers in adjusting liquid transport behaviors, overcoming the limitations of traditional strategies using fixed structures.

Moreover, gradient magnetic fields can be applied to reconfigure rectifiers with more complex and localized features, creating unprecedented opportunities for liquid manipulation. We discover a novel pulse‐type directional spreading consisting of four stages in the reconfigured rectifier under gradient magnetic fields constructed by double‐layer type‐2 magnets (Figure 4E–i; Movie S2, Supporting Information). The injected liquid (χ=100%) first fills the microratchet gaps in the straight‐through mode (stage ②). Then, the liquid is pinned by the maximum *X*+ and *X*− bending microratchets (≈29 s, stage ②). The pinning on both sides will trap the liquid for tens of seconds and cause an increase in the liquid accumulation height locally (≈88 s, stage ③). The bottom curvature results in a smaller Laplace pressure resistance in the *X*‐ direction (|Δ*p*_*X*__1‐_| < |Δ*p*_*X*__1+_|, Figure 3B–i). When the accumulation height of the liquid is sufficient, it will preferentially break the pinning in the *X*‐ direction and cause a sudden release of excess liquid, leading to pulse‐type spreading that instantly breaks the pinning of multi‐column microratchets (≈89 s, stage ④). The number of spanned columns over time in the four stages is summarized in Figure 4E–ii. Extended from the straight‐through mode, the pulse‐type spreading will disappear and transition to the reentrant mode when the liquid's surface tension is higher. We note that the liquid's reentrant mode in the reconfigured rectifiers under gradient fields is determined by the liquid injection positions (Movie S2, Supporting Information). At position A where microratchets are bent toward it, the liquid (χ=0%) encounters small resistance on both sides and exhibits bidirectional reentrant spreading (state ②‐②, Figure 4F–i, ii). At position B where microratchets are bent away from it, the liquid encounters high resistance on both sides. Yet, the heterogeneous cross section of the microratchet along the *X*‐axis (curved and flat surfaces) creates a new resistance difference (Figure S18, Supporting Information) to direct the liquid toward the *X*+ direction (state ②‐②, Figure 4F–iii, iv). Therefore, complex gradient magnetic fields turn microratchet morphologies from spatially uniform structures into more complex, spatially varying structures, enabling multiple novel modes of liquid manipulation in 3D.

### 2.5 Versatile Rectifiers for Practical Applications

Finally, we leverage our 3D liquid manipulation paradigm to demonstrate versatile rectifiers in portable liquid purity testing, multi‐channel logical liquid transport, and automated chemical reactions.

#### 2.5.1 Portable Liquid Purity Testing

**Figure 5:**
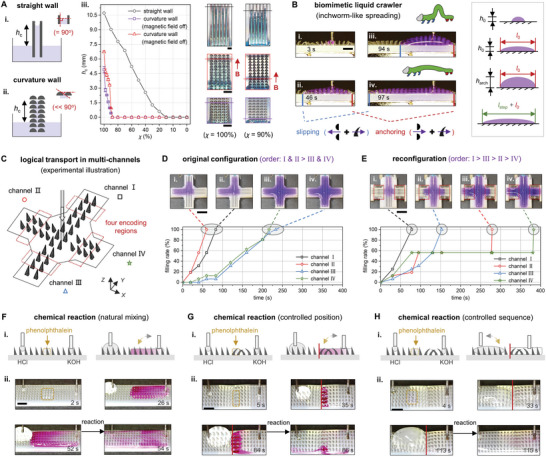
Versatile applications of the rectifier based on the proposed liquid manipulation paradigm. A) Abnormal capillarity used for liquid purity identification: i, ii) mechanism illustration of critical liquid contact angles in a continuous straight wall versus the curvature wall formed by the rectifier. iii) Quantitative comparison of rectifier's capillarity with conventional capillarity. B) 3D biomimetic liquid crawler: i–iv) different stages of the liquid spreading behavior and corresponding morphological parameter definitions. C) Illustration of the experimental setup for logical liquid transport in multiple open channels. D) Liquid transport in the original configuration of multi‐channels: i–iv) experimental snapshots of the moment when each channel is first filled. E) Liquid transport in the reconfigured multi‐channels follows a modified filling order of four channels: i–iv) experimental snapshots of the moment when each channel is first filled. F) Chemical reactions enabled by original configuration rectifier mediated natural mixing: i) illustration and ii) experimental snapshots. G) Chemical reactions with controlled position enabled by the reconfigured rectifier: i) illustration and ii) experimental snapshots. H) Chemical reactions with controlled sequence enabled by the reconfigured rectifier: i) illustration and ii) experimental snapshots. Scale bars: 1 mm (A), 2 mm (B), 10 mm (D, E), and 3 mm (F–H).

In our rectifier, we observe abnormal discrete variations in the capillary height (*h*_c_) for different ethanol‐water mixtures (**Figure** **5**A). In the capillarity phenomenon, *h*_c_ is mainly characterized by the liquid–air interface at the meniscus. A concave liquid–air interface means the additional capillary pressure can pull the liquid upward, resulting in *h*_c_ >0; a flat liquid–air interface implies no extra force, thus *h*_c_ = 0; a convex liquid–air interface indicates that capillary pressure hinders liquid rise and makes *h*_c_ <0. Based on this criterion, *h*_c_ is expected to decrease continuously with increased liquid contact angle. However, our rectifier exhibits a surprising, cliff‐like drop in *h*_c_, reaching near‐zero values at χ=90%. This observed behavior shows a binary trend, where *h*_c_ > 0 for χ≥90%, but *h*_c_ = 0 for χ<90%. This is due to the unique microratchet bottom curvature forming a curvature‐wall capillary channel, unlike the traditional straight‐wall design (Figure 5A–i,‐ii). The critical contact angle for the curvature‐wall channel to satisfy *h*_c_ = 0 is much smaller than that in the straight‐wall design (90°). This leads to a rapid downward trend in *h*_c_ (Figure 5A–iii), compared to the continuous variations in straight‐wall channels with the same normalized channel width (Figure S19, Supporting Information). The discontinuous curvature‐wall channels show a lower *h*_c_ than continuous straight‐wall capillary channels, and this capillary height loss can be compensated to an extent under magnetic fields (Figure 5A–iii; Figure S19, Supporting Information). From χ=100% liquid (≈22.89 mN m^−1^) to χ=90% liquid (≈23.82 mN m^−1^), the rectifier exhibits visible differences in *h*_c_ with surface tension difference below 1 mN m^−1^. Such a high resolution makes us believe its potential for rapid purity screening of low surface tension organic liquids. In contrast to current testing instruments with extra electronic or optical components, we provide a simple liquid purity testing method for separating qualified/unqualified liquids via the rectifier's interfacial phenomena (Figure S22, Supporting Information).

#### 2.5.2 Logical Liquid Transport in Multiple Open‐Channels

Based on the pulse‐type liquid spreading (under two‐layer type‐2 gradient magnetic fields, Figure 4E–i), we develop inchworm‐like 3D liquid regulation with logical switches that enable logical liquid transport in multiple open‐channels (Figure 5B; Figure S20 and Movie S3, Supporting Information). A ‘liquid crawler’ is formed within ≈46 s by injecting the χ=90% liquid (Figure 5B–i, ii). The crawler's initial height (*h*_0_) and length (*l*_0_) are determined by the liquid surface tension and the width of the magnet below, respectively. Pinned by bending microratchets on two sides, the liquid accumulates along the *Z*‐axis, resembling an inchworm's arched body (≈94 s, Figure 5B–iii). The critical height (*h*_arch_) is determined by the combined effect of magnetic fields and liquid surface tension. The liquid crawler preferentially breaks the pinning in the *X*‐ direction to exhibit pulse‐type liquid spreading (≈97 s, Figure 5B–iv), reminiscent of an inchworm extending its body by extending the distance between its anchoring position (red) and slipping position (blue). The crawling step length (*l*_step_) depends on the neighboring magnet's width. The 3D liquid crawler can be adjusted by changing liquid surface tension, magnet sizes, and arrangements (Figure S20, Supporting Information). This liquid crawler can be used as a logic switch for determining the space and time for liquid spreading in a multi‐channel rectifier.

Based on a reconfigurable four‐channel rectifier with localized magnet fields under each channel, we show the adjustable liquid filling sequence in channels I–IV through the logical switches described above (Figure 5C; Movie S4, Supporting Information). In the original configuration, the rectifier allows low surface tension liquids to spread toward the *X*− and *Y*+ directions (Figure S21, Supporting Information). Upon injecting the liquid (χ=100%) into the rectifier center, it fills channel I and channel II at ≈60 and ≈83 s (Figure 5D–i, ii). By disrupting the reverse structural pinning, the excess liquid proceeds to fill channels III and IV at ≈216 and ≈233 s (Figure 5D–iii, iv). By strategically configuring the magnetic fields, we can customize the filling order of channels on demand. For example, we reconstruct liquid filling priorities in four channels by arranging gradient magnetic fields (two‐layer type‐3 magnets, marked with red boxes) beneath channels II and IV (Figure 5E; Figure S21 and Movie S4, Supporting Information). With the obstruction in channel II, channel I holds the least pinning and is assigned the highest priority for filling at ≈78 s (Figure 5E–i). Because of substantial pinning caused by strongly curved ratchets in channels II and IV, channel III is changed to be the second priority to be filled (≈153 s, Figure 5E–ii). The directionality of the liquid crawler dictates that channel II will be filled first (≈279 s, Figure 5E–iii), while channel IV will be filled lastly (≈383 s, Figure 5E–iv). By incorporating modular magnetic fields, reconfigurable multi‐channel rectifiers may provide a potential approach for complex liquid operations in open‐channel microfluidics, such as multi‐step biochemical diagnostics (Figure S22, Supporting Information).

#### 2.5.3 Spatiotemporally Controlled Chemical Reaction Platforms

Spatiotemporal control of multiple liquids is crucial in chemical reactions and diagnostic analysis. Here we demonstrate how our rectifier, via precise 3D liquid manipulation, can enable such control in chemical reactions between potassium hydroxide (KOH), hydrochloric acid (HCl), and phenolphthalein solution (Experimental Section and Movie S5, Supporting Information). Figure 5F–i, ii shows the natural mixing of acid, base, and pH‐indicator. KOH and HCl move in opposite directions due to different surface tension, and mix with the middle pH‐indicator phenolphthalein (≈16 µL, marked in yellow). Low surface tension KOH rapidly spreads and displays color upon encountering the phenolphthalein (≈26 s). Then, the KOH‐phenolphthalein mixture spreads and initiates a chemical reaction upon encountering HCl (≈52 s). The surface tension difference between KOH and HCl expedites the mixing reaction, resulting in rapid fading of the solutions (≈54 s).

By utilizing the reconfigured rectifier under gradient magnetic fields (two‐layer type‐3 magnets), we demonstrate control over the position and sequence of chemical reactions. When applying the gradient magnetic field beneath phenolphthalein (Figure 5G–i), KOH is pinned upon encountering the pH‐indicator and moves in the opposite direction (≈35 s, Figure 5G–ii). Therefore, the indicator does not diffuse throughout the entire KOH but instead exhibits color only at the contact position. The reaction location is controlled at the pinning position. After HCl comes into contact with KOH (≈84 s), the chemical reaction is triggered (≈86 s). Similarly, by adjusting the pinning point between phenolphthalein and KOH (Figure 5H–i), we can control the preferential reaction sequence (Figure 5H–ii). In this case, HCl and phenolphthalein react first (≈33 s) as KOH is strongly pinned. Then, it is followed by the subsequent acid‐base reaction (≈113–115 s). Due to pre‐mixing of HCl and phenolphthalein, the color development during this rapid reaction is hardly observable. With pre‐customized magnetic fields, this spatiotemporally controllable liquid manipulation paradigm enables non‐experts to easily implement preset liquid operations, potentially serving as point‐of‐care devices (Figure S22, Supporting Information).

## 3 Conclusion

In this study, we propose a novel liquid manipulation paradigm that combines multimodal liquid rectifiers and programmable magnetic fields. The tightly integrated structural design and external field actuation are unified into adjustable interfacial energy to enable on‐demand 3D liquid manipulation. The novelty of this liquid manipulation strategy arises from the rectifier's high‐performance directing capability, wide adjustability, as well as the simplicity and scalability of the magnetic field intervention scheme. The combination of high‐performance rectifiers and customized magnetic fields in our new paradigm mitigates the long‐standing trade‐off between the simplicity of structured surfaces and the flexibility of external‐field‐controlled liquid manipulation. Furthermore, this reconfigurable system can enable static rectifiers to achieve novel biomimetic 3D liquid regulation as well as controlling spatiotemporal behaviors of liquid transport. These advancements pioneer new applications in portable liquid identification, open‐channel microfluidics, point‐of‐care devices, and biomedical applications. From a broad perspective, the proposed liquid manipulation paradigm can be extended to coupling methods involving various external fields and surface structures, and be considered to develop more complex multiphase interfacial operations.

## 4 Experimental Section

### Materials

Ethanol (AR, water ≤0.3%), isopropyl alcohol (ACS, ≥99.5%), potassium hydroxide (ACS), hydrochloric acid (ACS, 37%), phenolphthalein (98%) were provided by Aladdin Industrial Co., Ltd. (Shanghai, China). The silicon elastomer Ecoflex 00‐30 was purchased from Smooth‐on Inc. (Macungie, PA, USA). Iron microparticles (≥99.9%, with an average diameter of 1 µm) were obtained from the Leber Inc. (Beijing, China). The ferrite magnet (150 mm × 100 mm × 25 mm, used for magnetic‐field‐assisted molding process), type 1–3 NdFeB magnets (N52), and cotton swabs were obtained from standard retailers in China. The used dyes, including violet biodye solution (2.5%) and methylene blue (MB) dye (AR), were purchased from BKMAM Biotechnology Co., Ltd. (Changde, China). Deionized water (DI water) was produced by the Direct‐Q deionized water system (Millipore, MA, USA).

### Fabrication of the Rectifier

The rectifier was prepared via a magnetic‐field‐assisted molding process. The 3D‐printed mold was manufactured via the projection micro stereolithograph (equipment: BMF MicroArch S240; printing resolution: 10 µm). Before the molding process, the mold was sonicated in isopropyl alcohol for 15 min and then treated with oxygen plasma for 30 min using a plasma cleaner from Harrick Plasma Inc. This treatment aimed to eliminate mold impurities and prevent them from affecting the silicone elastomer curing process. Ecoflex 00‐30, a platinum‐catalyzed silicone elastomer, was used as the main material of the rectifier. A non‐magnetic precursor was prepared (Ecoflex A and Ecoflex B in a mass ratio of 1:1) for the rectifier's substrate and magnetic precursor (Ecoflex A, Ecoflex B, and iron microparticles in a mass ratio of 1:1:1) for the rectifier's microratchets. The magnetic precursor was first poured into the mold and placed in a vacuum environment for 10 min to remove air bubbles. Afterward, the upper layer of the magnetic precursor was carefully wiped off using cotton swabs, then it was replaced by the non‐magnetic precursor. Subsequently, the mold was positioned above a ferrite magnet (150 mm × 100 mm × 25 mm) during the curing process. This large‐sized magnet ensured vertical magnetic field lines to align soft‐magnetic microparticles (Figure S4A, Supporting Information). The magnetic field strength was ≈120 mT measured by a Gaussmeter (resolution: ±2%, Nohawk, Inc. Tianjin, China). The covered non‐magnetic precursor prevented the Rosensweig‐type instability, ensuring solidification stability. Finally, the rectifier was obtained by demolding after curing for 12 h at room temperature (25 °C).

### Microscopy

The reconstructed 3D surface topography of the rectifier (layer thickness: 10 µm) was imaged using a 3D Laser Scanning Microscope (VK‐X200; Keyence, Osaka, Japan). The SEM images of the microratchets were observed using a Hitachi S‐3400N scanning electron microscope.

### Vibrating Sample Magnetometry

Vibrating sample magnetometry (VSM) data were taken at 300 K in the range from –20 000 (–2 T) to +20 000 Oe (+2.0 T) measured by a vibration magnetometer (8600 Series VSM, Lake Shore Cryotronics, Inc. OH, USA).

### FEA Simulation of Magnetic Fields

A 2D simulation using the finite element analysis (FEA) software MAXWELL was applied to study the magnetic field distribution of the programmed magnet arrays. The selected magnet material was N52, which was the same model as the purchased NdFeB magnets. The magnet arrangement in the simulated magnetic field is shown in Figure S6 (Supporting Information). The magnetic field line vector distribution and magnetic field intensity distribution of different magnetic fields were simulated separately, as illustrated in Figures S7,S8 (Supporting Information).

### Experimental Setup

The liquid‐infused model described in the study was based on the experimental setup depicted in Figure S9A (Supporting Information). The setup included the following components: a syringe pump (LSP02–2B, Longer Pump), a syringe (capacity: 10 mL; inner diameter: 14.8 mm) connected to soft tubes, and a syringe needle (inner diameter: 0.3 mm), a 3‐DOF *XYZ* precision positioning platform (displacement range: ±6.5 mm along the *X*‐axis and *Y*‐axis, 10 mm along the *X*‐axis; resolution: 0.03 mm), NdFeB magnet arrays, a hand‐held microscope (Dino‐Lite Edge AM4115ZTL, AnMo, Taiwan) used in conjunction with Dino Capture 2.0 software, a spirit level and the rectifier. Different configurations of the rectifier were switchable by placing/removing the magnet arrays as required (Figure S9B, Supporting Information). The liquid injection flow rate was typically set at 130 µL min^−1^ unless stated otherwise. The distance between the injection needle and the rectifier could be accurately adjusted using the 3‐DOF positioning platform. The suitable injection height ranges of different surface tension liquids are shown in Figure S10 (Supporting Information). For liquids with low surface tension, the injection height was lowered to prevent the inertia effect caused by gravity; for liquids with high surface tension, the height was raised a little to avoid pinning the liquid with the needle. The data obtained from all experiments were processed using ImageJ software.

### Property Measurement of the Experimental Liquids

The measurements of surface tension, apparent contact angles, and advancing and receding angles for the experimental liquids were acquired using an optical video‐based contact angle meter (Model 100SB, Sindatek Instrument Co., Ltd., New Taipei City, Taiwan). The surface tensions of the experimental liquids (ethanol‐water mixture) were directly identified by the equipment supporting software (Figure S3A, Supporting Information). As the ethanol content (*χ*) decreased, the liquid surface tension increased. Apparent contact angles of experimental liquids on magnetic and non‐magnetic substrates (30 mm × 30 mm × 2 mm) were measured by recognizing a small droplet of liquid (≈5 µL) to evaluate the surface energy uniformity (Figure 2B–iv). For a fixed substrate material, the liquid apparent contact angle was positively related to its surface tension. In addition, the advancing and receding contact angles (*θ*_a_ and *θ*_r_) of the experimental liquids were measured by advancing and receding ≈5 µL liquid onto the non‐magnetic substrate (Figure S3B, Supporting Information). In experiments, the liquids were dyed in purple (χ≥50%, violet dye solution concentration in experimental liquids: 1 mg mL^−1^) and blue (χ<50%, methylene blue concentration in experimental liquids: 0.2 mg mL^−1^) to improve visualization. The added dyes were confirmed to have no significant effect on the surface energy of the liquid (Figure S3C, Supporting Information).

### Generality Evaluation Experiment of the Rectifier

Generality evaluation experiments include two approaches. One approach was to slightly change a certain size parameter to study the changed *We*‐*χ* phase diagrams; the other approach was to proportionally scale up/down the original rectifier. The rectifiers of various sizes were manufactured by modifying the design dimensions of the mold and the standardized magnetic‐field‐assisted molding process was implemented. The Weber number (*We*) was calculated according to Equation (11). The inner diameter of the syringe needle (*D*) was 0.3 mm. For a specific liquid, the density (*ρ*) and surface tension (*γ*) were fixed. Therefore, adjustable liquid injection speed could be directly converted into the corresponding *We* (Figure 3F). To investigate the scale effect, a scale coefficient was defined to describe rectifiers with different scales (Figure S15, Supporting Information). Under the rectifiers of various scales, the spreading modes of the experimental liquids were re‐examined (including *X*− and *X*+ directional spreading, and bidirectional spreading) and the phase diagrams of the spreading modes were provided (Figure 3G).

### Preparation of Chemicals

To showcase the rectifier's multi‐liquid operation capabilities, spatiotemporally controlled chemical reactions were performed as illustrative examples. The chemicals were prepared with standardized concentrations, including ethanol‐based potassium hydroxide solution (KOH, 1 M L^−1^), aqueous hydrochloric acid solution (HCl, 1 M L^−1^), and ethanol‐based phenolphthalein solution (5 g L^−1^). Specifically, 0.56 g of KOH was added to 10 mL of ethanol to make the 1 M L^−1^ KOH solution; 1 mL of 37% HCl was mixed into 11 mL of DI water to prepare the 1 M L^−1^ HCl solution; 0.05 g of phenolphthalein was dissolved in 10 mL of ethanol to prepare the 5 g L^−1^ phenolphthalein solution. By using different solvents, KOH, and phenolphthalein solutions had lower surface tensions than the HCl solution.

## Conflict of Interest

The authors declare no conflict of interest.

## Supporting information

Supporting Information

Supplemental Movie 1

Supplemental Movie 2

Supplemental Movie 3

Supplemental Movie 4

Supplemental Movie 5

## Data Availability

The data that support the findings of this study are available from the corresponding author upon reasonable request.
