# Supplementary material for: Reconfigurability‐Encoded Hierarchical Rectifiers for Versatile 3D Liquid Manipulation
Source: Adv Sci (Weinh). 2024 Jul 29;11(39):2405641. doi: 10.1002/advs.202405641 (PMC11497013; doi:10.1002/advs.202405641)
Supplement: Supplementary file 1 — Supporting Information [file ADVS-11-2405641-s003.pdf]

ADVANCED SCIENCE
Open Access

Supporting Information

for *Adv. Sci.*, DOI 10.1002/advs.202405641

Reconfigurability-Encoded Hierarchical Rectifiers for Versatile 3D Liquid Manipulation

*Jiaqi Miao and Alan C. H. Tsang**

Supporting Information for "Reconfigurability-Encoded Hierarchical Rectifiers for Versatile 3D Liquid Manipulation"

Jiaqi Miao[1], Alan C. H. Tsang[1*]

This PDF file includes:

Supplementary Sections I-XII

I. 3D Printing Setup and Customizable Rectifier Design

II. Property Masurement of The Experimental Liquids

III. Magnetic-Field Driven Microratchet Bending

IV. Construction and Simulation of The Magnetic Fields

V. Experimental Setup

VI. Mechanism Analysis of The Two Main Directional Spreading Modes

VII. Multimodal Spreading Modes and The Generality Evaluation

VIII. Reentrant Spreading Mode Under The Reconfigured Rectifiers

IX. Capillarity-Mediated Liquid Purity Testing

X. 3D Liquid Crawler with Adjustable Morphological Parameters

XI. Logical Liquid Transport in Multi-Channels

XII. Summary of Potential Applications

Figures S1-S22

Legends for Movies S1-S5

* [1] Department of Mechanical Engineering, The University of Hong Kong, Hong Kong. alancht@hku.hk

I. 3D PRINTING SETUP AND CUSTOMIZABLE RECTIFIER DESIGN

The detailed dimensions of the 3D-printed mold are provided in Figure S1Ai. Both the upper substrate part and the microratchet part have a height of 1200 µm. To account for printing accuracy, we designed and processed a bottom semicircular structure with a diameter (R_0) of 30 microns. Therefore, the molded microratchet will have a cone tip structure. To reduce printing time and ensure high accuracy of the mold's dimensions, we employed different slicing layer thicknesses in three parts of the mold (Figure S1Aii). Specifically, the top structure (part 1) and bottom mold support (part 3) have a slicing thickness of 40 µm, and the middle part 2 has a slicing thickness of 10 µm. During the actual printing process, the fast printing part (with a 40-µm layer thickness structure) is subjected to longer UV exposure time and high light intensity to ensure enough mechanical strength (Figure S1Aiii). Additionally, the resin leveling time (i.e., delay time) between different layers is reduced to expedite the printing process. We employed the opposite approach to ensure the high processing accuracy of the middle (part 2). We used a shorter UV exposure time, lower light intensity, and increased delay time to allow for meticulous layer-by-layer curing. The parameter setting of the printing processes for the three parts of the mold is summarized in Figure S1Aiii. The 3D printing platform, as depicted in Figure S1B, comprises the following components: a 3D printer, a personal computer (PC), and a supporting table. Supported by this high-resolution 3D printing, we can achieve on-demand designs for the functional rectifiers (Figure S1C). The final products combine on-demand rectifier design, programmed external magnetic fields, and other accessory components to fulfill diverse liquid manipulation requirements.

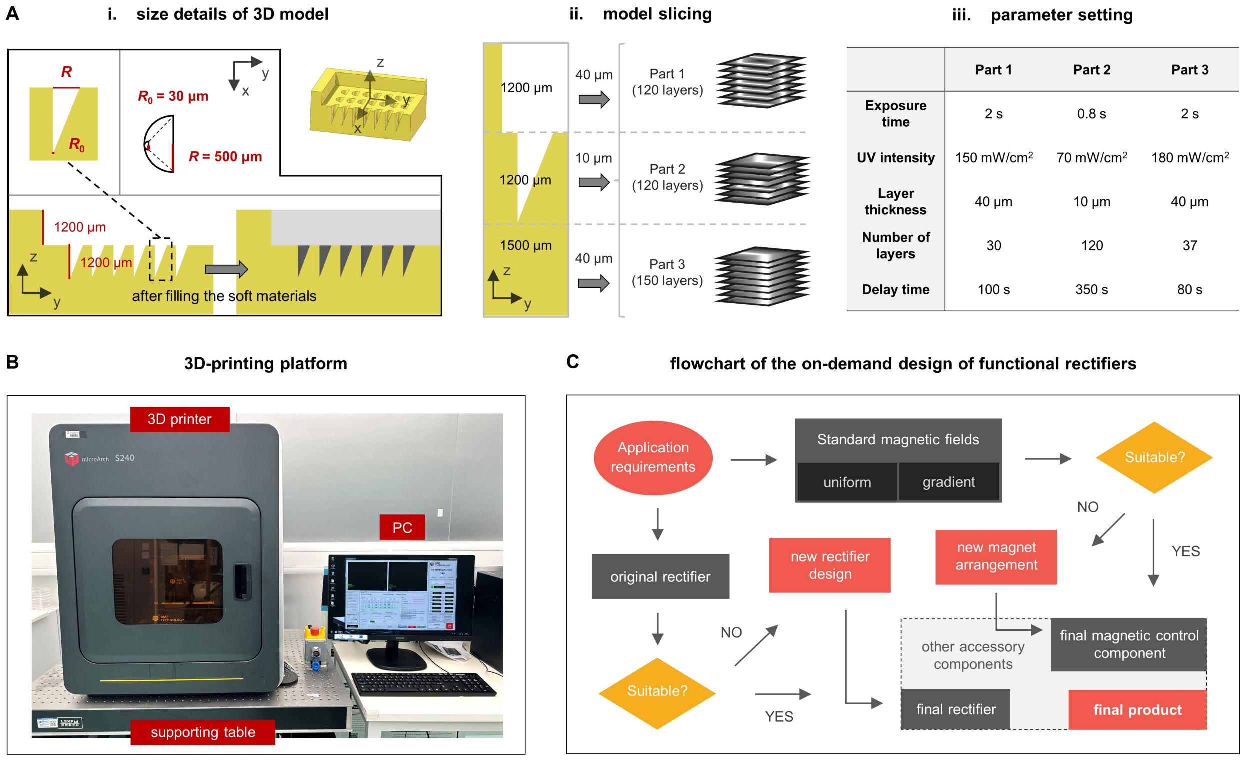

FIG. S1. 3D printing for customizable rectifier design. (A) Illustration of (i) the size details of the mold; (ii) mold slicing; and (iii) printing parameter setting. (B) 3D printing setup. (C) Flowchart of the customizable rectifier for liquid manipulation.

In Figure S2, we show the measured size parameters (R, h, d_v, and d_h) of the molded microratchets (according to their SEM images), and compare them with the design values. The actual size values closely match the design values in our customized magnetic-field-assisted molding process. The slightly larger difference in h (mean ± SD: 973.8 ± 35.4 µm) comes from the instability of the tip structure during the molding process, leading to an average error of ∼ 226.2 µm from the design value (1200 µm). Moreover, we show the full view of the rectifier placed in a Petri dish (as the image shown in Figure S2).

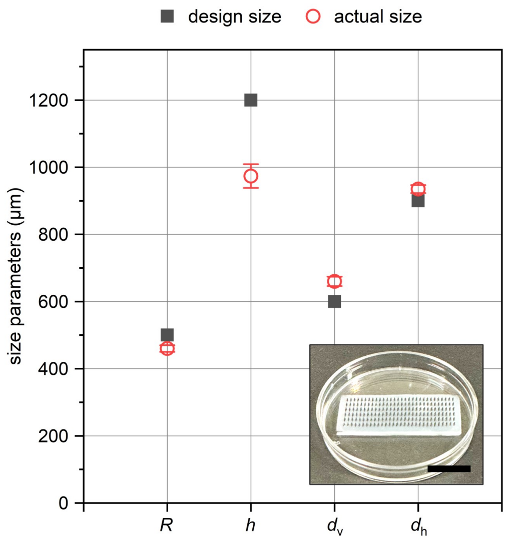

FIG. S2. Comparison of the design values and actual values of the rectifier's size parameters, embedded with the image of the rectifier in a Petri dish. Error bars denote the SD. Scale bar: 10 mm.

II. PROPERTY MEASUREMENT OF THE EXPERIMENTAL LIQUIDS

Figure S3 presents the experimental results obtained using the liquid property measurement method described in the Experimental Section of the main text. As χ decreases from 100% to 0%, the surface tension of the binary liquid mixture correspondingly increases (Figure S3A), transitioning from pure ethanol (mean: 22.89 mN/m) to pure water (mean: 71.97 mN/m). Considering this nonlinear relationship, we made appropriate adjustments to the liquid parameter settings during the experiments. Specifically, we increased the set point density within the range of 20% to 0% (with an interval of 5%). However, for the set point density within the range of 100% to 20%, the interval was set at 10%. In Figure S3B, the advancing, static, and receding contact angles of the binary mixture are presented. It was observed that for the same liquid, the advancing contact angle is greater than the static contact angle, while the receding contact angle is smaller than the static contact angle. To assess the impact of the added dye, we compared the contact angles of the $\chi = 60\%$ liquid (on the non-magnetic substrate material: Ecoflex) in the following scenarios (Figure S3C): (i) without any dyes, (ii) with the addition of methylene blue dye, and (iii) with the addition of violet biodye solution. The similar contact angles (56.0°, 56.5°, and 57.6°) observed in Figure S3Ci-iii indicate the negligible effect of the dye on the surface energy of the liquid.

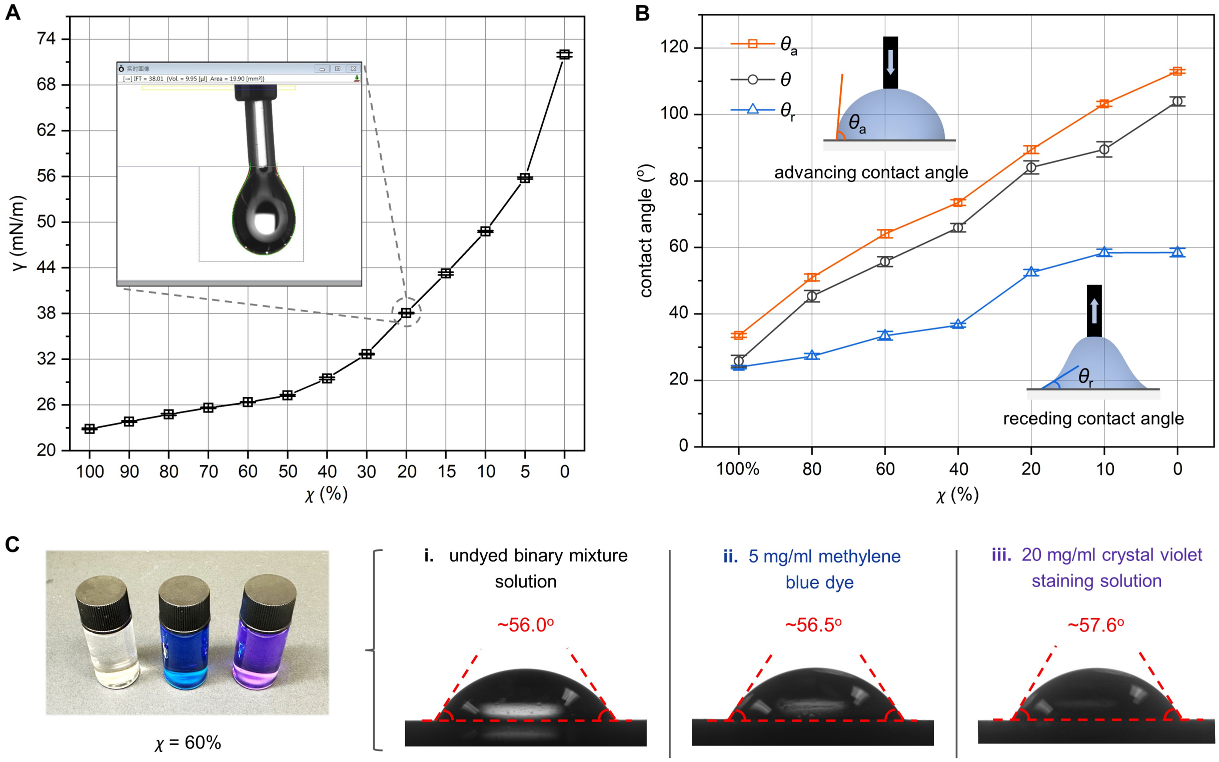

FIG. S3. Property measurement of the experimental liquids. (A) The surface tension of the liquid with different χ. (B) Advancing/static/receding contact angle of the liquid with different χ. (C) Contact angle comparison of the liquids (i) without dye, (ii) with the methylene blue dye; (iii) with the violet biodye.

III. MAGNETIC-FIELD DRIVEN MICRORATCHET BENDING

The bending properties of the microratchet are related to the used magnetic material types. Therefore, it is necessary to distinguish typical hard-magnetic and soft-magnetic materials. As illustrated in Figure S4Ai, hard-magnetic materials (such as NdFeB microparticles) are characterized by a large hysteresis due to high coercivity and remanence. Under a strong external magnetic field, hard-magnetic microparticles can be magnetized and endowed with an intrinsic magnetization direction. By contrast, soft-magnetic materials (e.g., iron microparticles) have a small hysteresis due to low coercivity and remanence (Figure S4Aii). When exposed to magnetic fields, iron microparticles will arrange along the magnetic field lines and thus acquire an intrinsic "magnetization" direction. In our design, each microratchet was endowed with such an intrinsic arrangement direction of magnetic microparticles (Figure S4Bi). When the microratchet is exposed to a magnetic field, its magnetic alignment axis inclines to bend towards the direction of the local magnetic field lines, regardless of the magnetic field direction (Figure S4Bii). A simple and intuitive example is the microratchet bending pattern under the standardized gradient fields (Figure 2D of the main text). In Figure S4C, it is observed that three adjacent magnets create two magnetic field regions above them (indicated by red dotted boxes). These two regions exhibit an identical distribution of magnetic field lines but in opposite directions. Based on our microratchet bending criterion mentioned above, i.e., the bending follows the nearest parallel to the magnetic field lines regardless of the direction of the magnetic field, therefore the microratchet bending pattern in the two regions is completely consistent. Figure S4D demonstrates how the orientation of magnetic field lines at various angles (α_B) influences the clockwise bending (referred to as $X+$ bending in the main text) and counterclockwise bending (referred to as X- bending in the main text) of the microratchet.

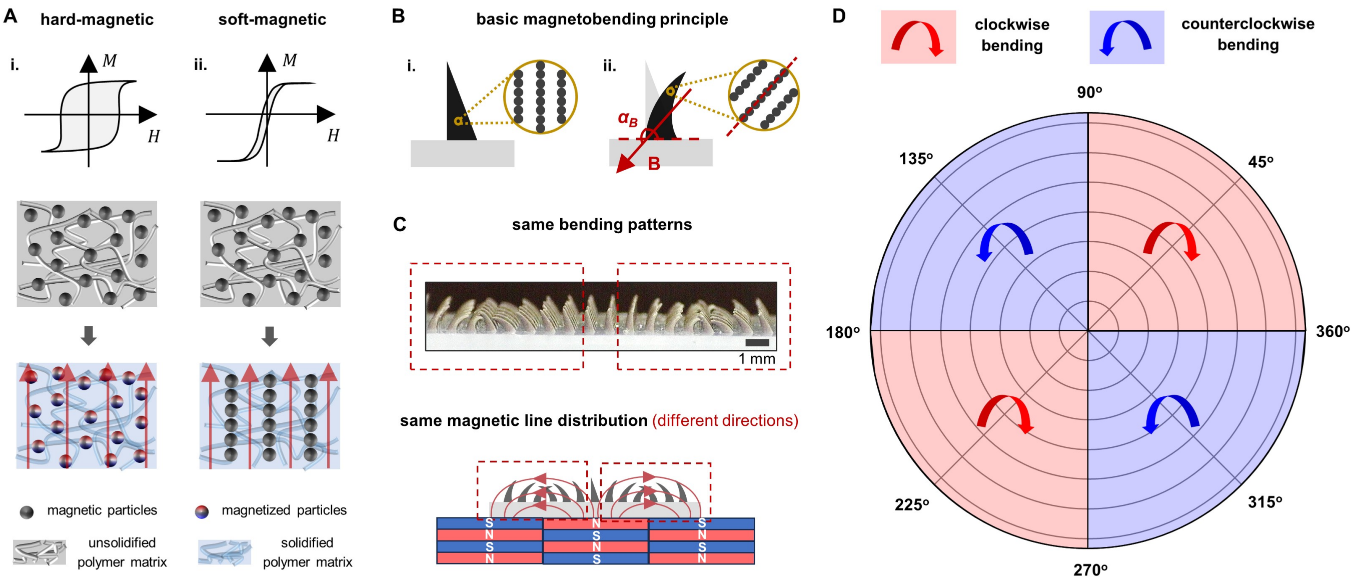

FIG. S4. Magnetic-actuated microratchet bending. (A) Illustration of the magnetization difference between (i) hard-magnetic materials and (ii) soft-magnetic materials. (B) Illustration of (i) the intrinsic magnetic particle arrangement direction and (ii) the basic magnetic-driven bending principle. (C) The magnetic field line distribution of gradient magnetic fields and the corresponding microratchet bending patterns. (D) Clockwise/counterclockwise bending under different α_B.

A key feature of microratchet bending is bending insufficiency. This phenomenon is more obvious in cases where a large bending angle is required to align the microratchet with the magnetic field lines. Figure S5A illustrates how this insufficient bending occurs in the $X+$ bending with a small α_B. This bending insufficiency stems from the limited magnetic field torque and force exerted on soft magnetic materials. Moreover, the bending of the materials is constrained by their inherent elasticity. Taking the constructed uniform magnetic field as an example (Figure 2C of the main text), we illustrate this bending insufficiency feature in Figure S5B. Due to the inherent elastic limitation, the microratchet cannot bend completely parallel to the magnetic field lines. Although this situation can be improved by increasing the magnetic field strength, such as transitioning from a type-2 uniform magnetic field (Figure S5Bi) to a type-1 uniform magnetic field (Figure S5Bii), insufficient bending can not be fully eliminated.

Moreover, there is a notable heterogeneity in the bending behavior, that is, the top structure can achieve full bending while the lower portion lacks sufficient bending (Figure S5C). We use a simple cantilever beam bending model to analyze this difference. Ignoring the difference in magnetic field strength under small height changes, the uniformly distributed magnetic particles cause the load on the microratchet to be uniform. Compared to the bottom structure, the second moment of area (I_y) in the top structure has a value close to 0, calculated based on the equation $I_y = 0.10976R^4_{\text{bottom}}$ [1]. Also, the top structure is positioned further away from the fixed end. This leads to the observed heterogeneity in curvature, with higher curvature at the top and lower curvature at the bottom of the structure. Figure S5D shows the bending microratchet under the type-2 and type-1 uniform magnetic fields. Utilizing the curvature heterogeneity property, the slight $X+$ bending microratchet can exhibit curvature at the top and nearly no curvature at the bottom (Figure S5Di), which forms the polar state of the rectifier (Figure 4B of the main text). In contrast, the more strongly bending microratchet will exhibit curvature at both the top and bottom (Figure S5Dii), consistent with the aggregation stage (Figure 4B of the main text). The curvature heterogeneity still exists, that is, the top structure is easier to bend, while the bottom structure shows stronger bending restriction.

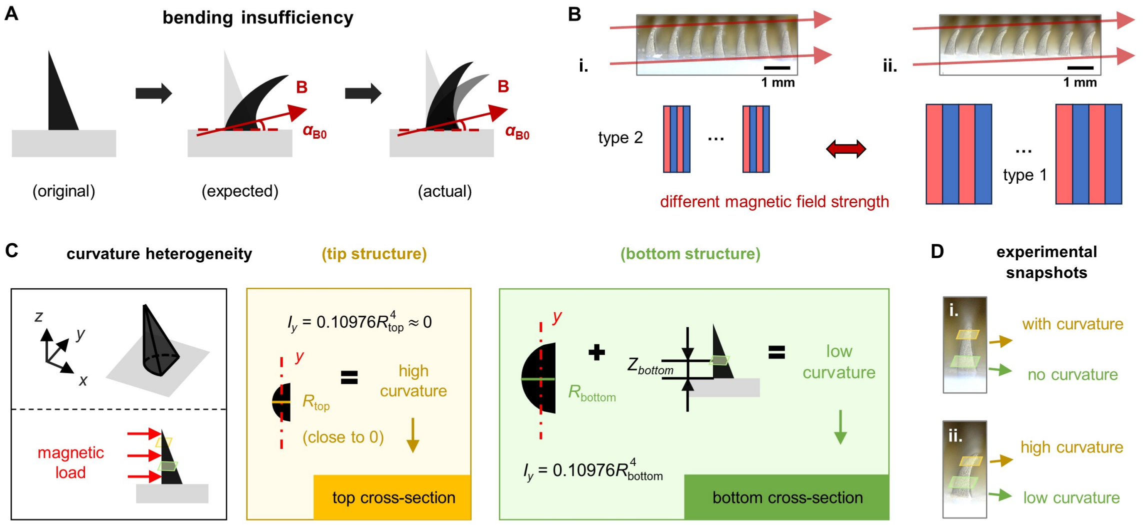

FIG. S5. Magnetic-actuated microratchet bending properties. (A) Illustration of the bending insufficiency. (B) The insufficient bending microratchets under (i) type-1 and (ii) type-2 uniform magnetic fields. (C) Curvature heterogeneity of tip and bottom structures. (D) The curvature heterogeneity of bending microratchets under (i) type-1 and (ii) type-2 uniform magnetic fields.

IV. CONSTRUCTION AND SIMULATION OF THE MAGNETIC FIELDS

The magnetic fields utilized in this study consist of small-volume NdFeB magnet arrays. For illustrative purposes, we employ three different sizes of magnets, referred to as type 1, type 2, and type 3. Their dimensions are shown in Figure S6A. Here we demonstrate the construction of magnetic fields according to the arrangement method described in Section 2.2 of the main text. As depicted in Figure S6B-C, we utilize the attractive forces between magnets to create different magnetic field configurations. Ultimately, we employ type 1, type 2, and type 3 magnets to construct three types of uniform magnetic fields and six types of gradient magnetic fields (derived from the one/double-layer stack of three types of magnets). Following the FEA simulation of magnetic fields outlined in the Experimental Section of the main text, we present the magnetic field vector and strength distribution for various magnetic fields in Figure S7-S8.

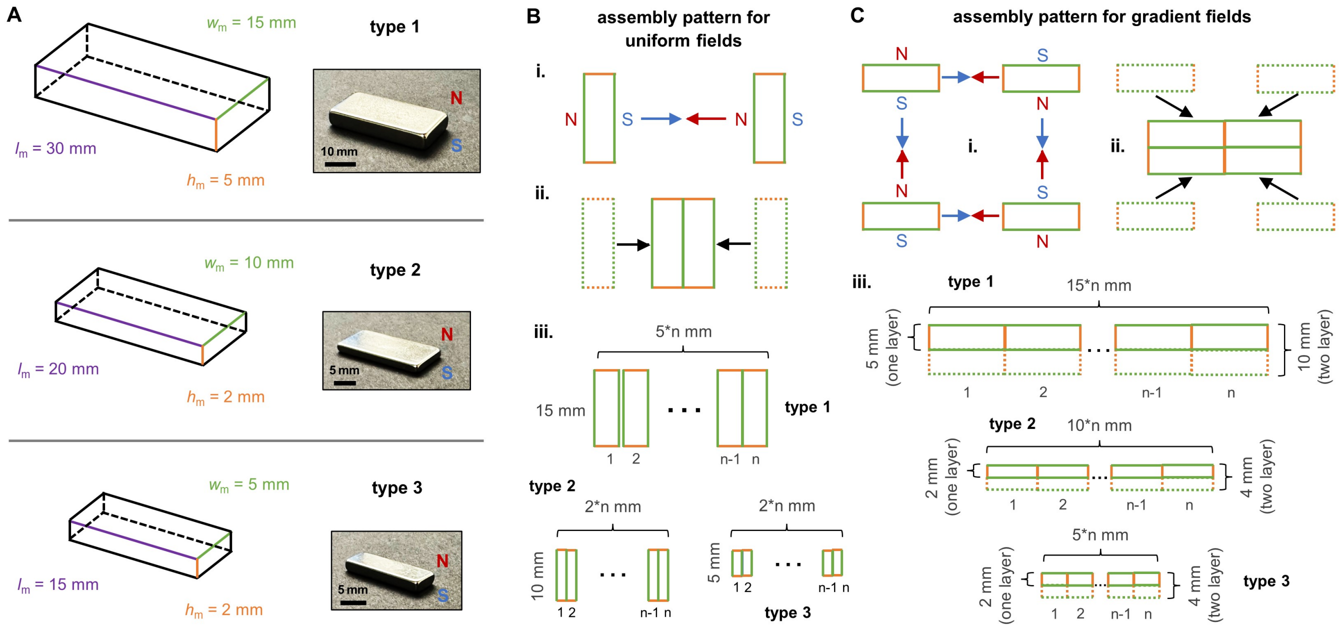

FIG. S6. Construction of magnetic fields. (A) Illustration of the three types of magnets. (B) Illustration of (i)-(ii) the construction method of the uniform fields and (iii) different assembly patterns. (B) Illustration of (i)-(ii) the construction method of the gradient fields and (iii) different assembly patterns.

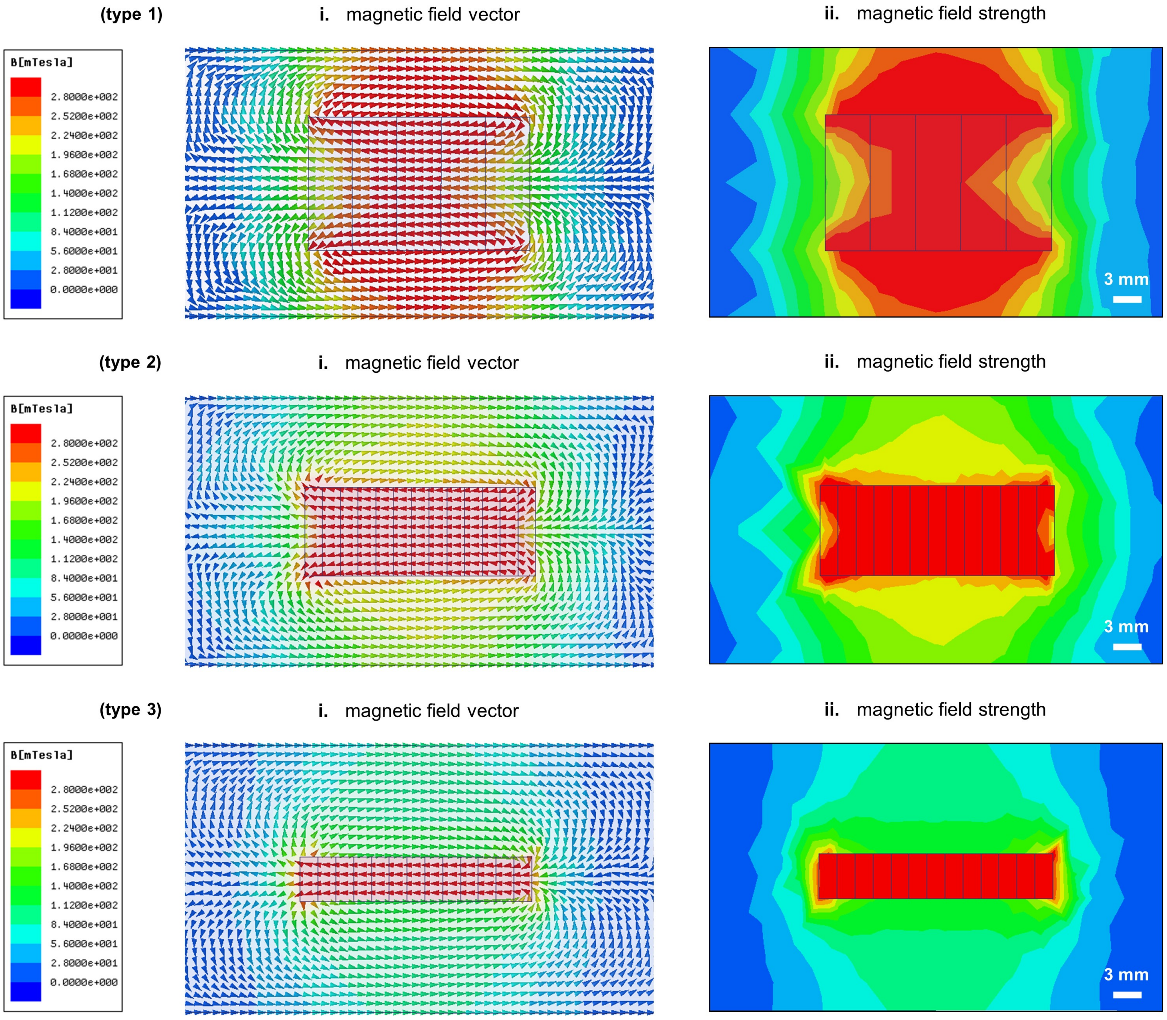

FIG. S7. Simulation of the constructed uniform magnetic fields.

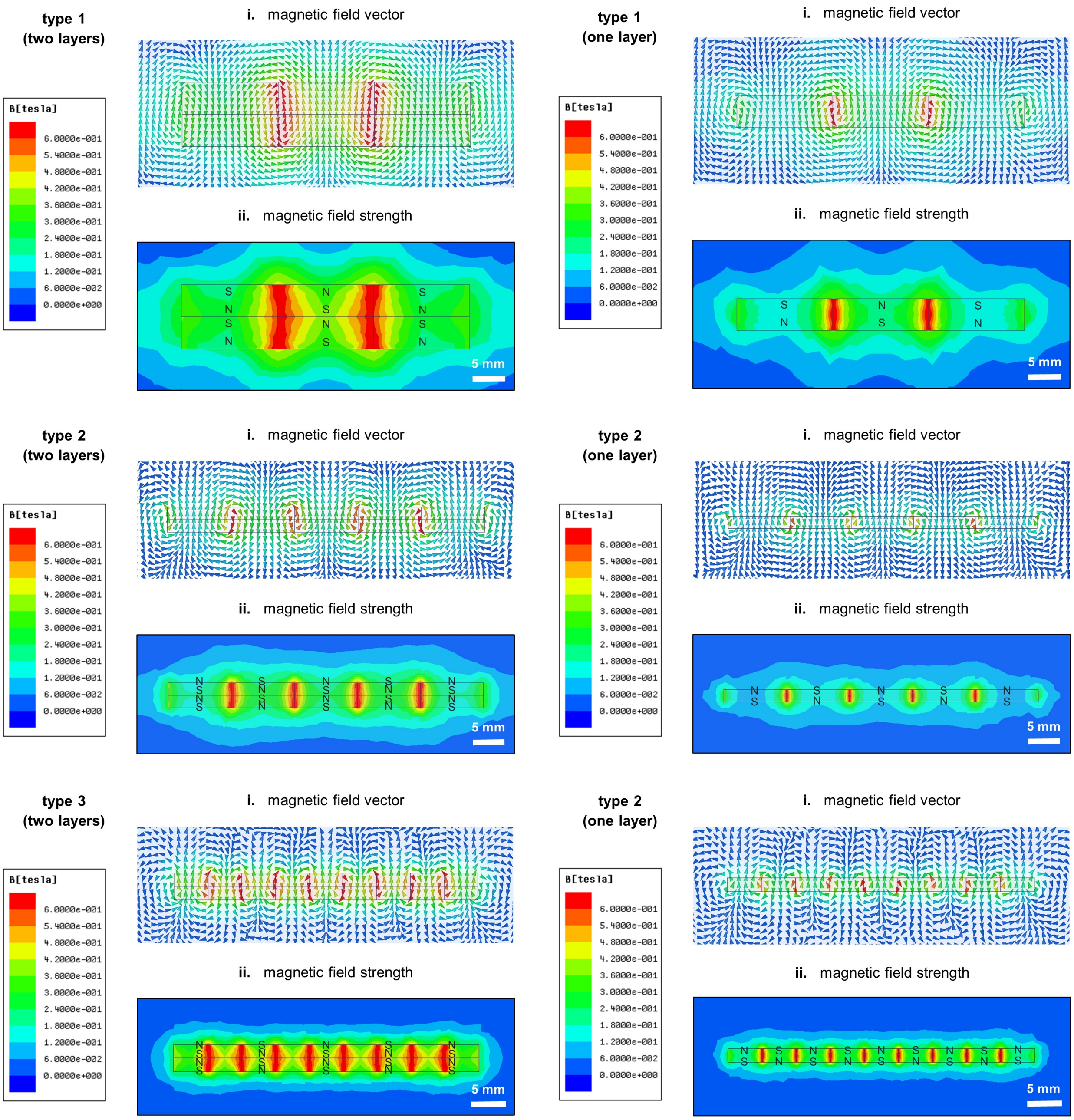

FIG. S8. Simulation of the constructed gradient magnetic fields.

V. EXPERIMENTAL SETUP

Figure S9A-B shows the experimental setup for investigating liquid spreading behaviors on reconfigurable rectifiers. Specifically, it contains a syringe pump, an iron support stand for fixed connecting tubes and needles, a handheld microscope with a fixture, a personal computer, a 3-DOF precision positioning platform, a spirit level, a rectifier, and arrayed magnets (if needed). Figure S10A-C shows the suitable liquid injection heights for different surface tension liquids, according to the descriptions in the Experimental Section of the main text.

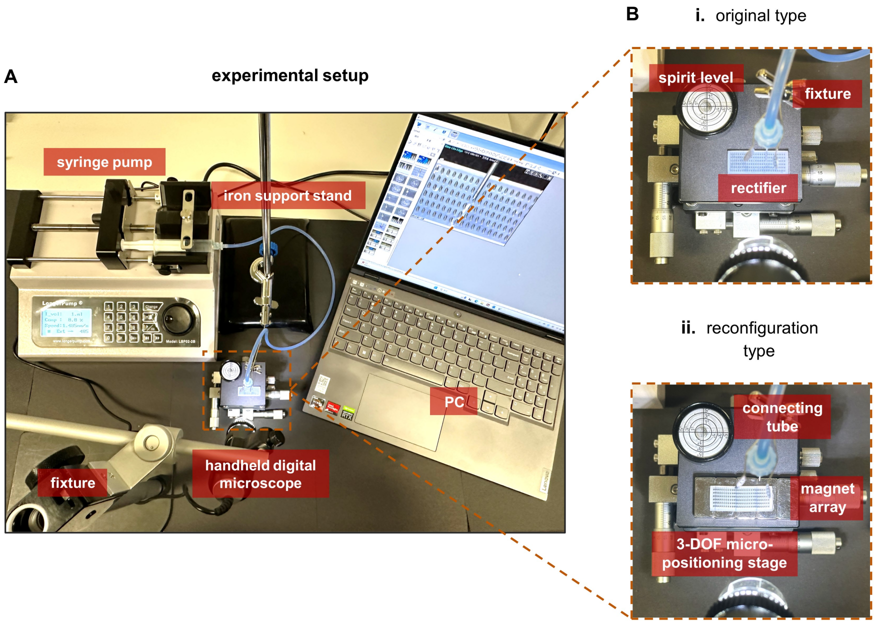

FIG. S9. (A) Illustration of the experimental setup. (B) Illustration of the experimental setup arrangement for (i) the original configuration rectifier and (ii) the reconfigured rectifier.

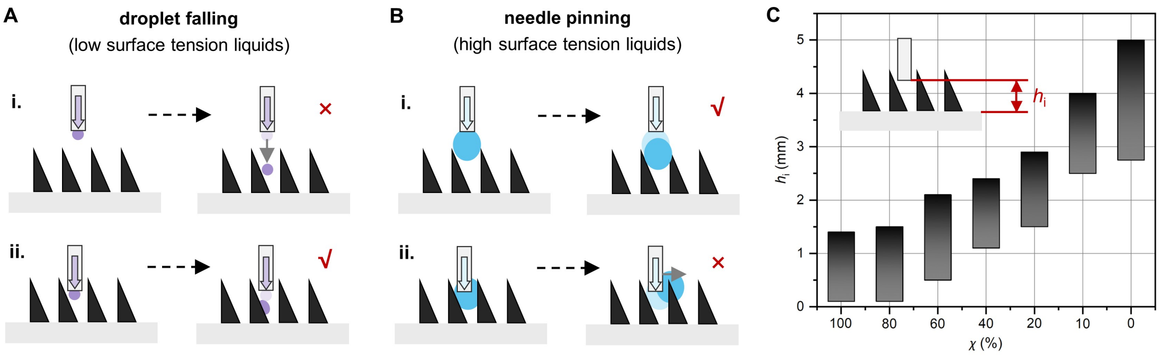

FIG. S10. (A) Mechanism illustration of how to choose suitable liquid injection height for the straight-through mode. (B) Mechanism illustration of how to choose suitable liquid injection height for the reentrant mode. (C) Suitable liquid injection height ranges for liquids with different ethanol concentrations.

VI. MECHANISM ANALYSIS OF THE TWO MAIN DIRECTIONAL SPREADING MODES

In this section, we supplement the detailed analysis of the two main liquid spreading modes, namely the straight-through mold and the reentrant mode. For the straight-through mode, we illustrate all the components of Laplace pressure in Figure S11. Based on the geometric relation in Figure S11A, $\Delta p_{X1\text{-}}$ and Δp_{X1+} can be readily obtained by using Equations (3)-(4) described in the main text. As for the Laplace pressure components $\Delta p_{X2\text{-}}$ and Δp_{X2+}, we give the detailed derivation via trigonometry based on the definitions in Figure S11B. In the $X+$ direction, Δp_{X2+} is directly determined by r_{X2+}, which is given by:

$$r_{X2+} = d_+/2\cos\beta_+, \tag{S1}$$

where d_+ and β_+ are the auxiliary parameters for calculating the curvature radius. They can be expressed as:

$$d_+ = h_{X+}/\sin\frac{\alpha}{2}; \quad \beta_+ = (180^{\text{o}} - \alpha + 2\theta)/2. \tag{S2}$$

When substituting Equation (S2) into Equation (S1), r_{X2+} is described as $h_{X+}/(2\sin\frac{\alpha}{2}\sin\frac{\alpha-2\theta}{2})$. Finally, we can get Equation (6) in the main text to evaluate the Laplace pressure component Δp_{X2+}. Similarly, we can calculate the Laplace pressure component $\Delta p_{X2\text{-}}$ based on Equation (5) in the main text. The only difference is that the structural angle changes from α to 90°. Due to $|\Delta p_{X1\text{-}}| < |\Delta p_{X1+}|$ and $|\Delta p_{X2\text{-}}| > |\Delta p_{X2+}|$, the total resistance from Laplace pressure in the X- direction ($\Delta p_{X\text{-}}$) is lower than that in the $X+$ direction (Δp_{X+}). When the injected liquid continuously expands the boundary, the pinning in the X- direction is preferentially broken, resulting in directional spreading.

In the reentrant spreading mode, the injected liquid undergoes the first entrant after accumulating a certain volume, then the liquid begins to spread towards X- direction in a continuous reentrant manner (Figure S11C). In addition to the mechanism description in the main text, the two side edges of the microratchet form a smaller structural angle with the substrate ($\alpha' = \arctan h/\sqrt{2}R$), which results in a smaller advancing contact angle $\theta'_{X+} = \min\{\theta_{\text{a0}} + \alpha', 180^{\text{o}}\}$. Based on the relationship $\theta'_{X+} \geq \theta_{X+} \geq \theta_{X\text{-}}$, the liquid on the two sides, which is more easily extensible, to pull the main body into a noticeable depression area.

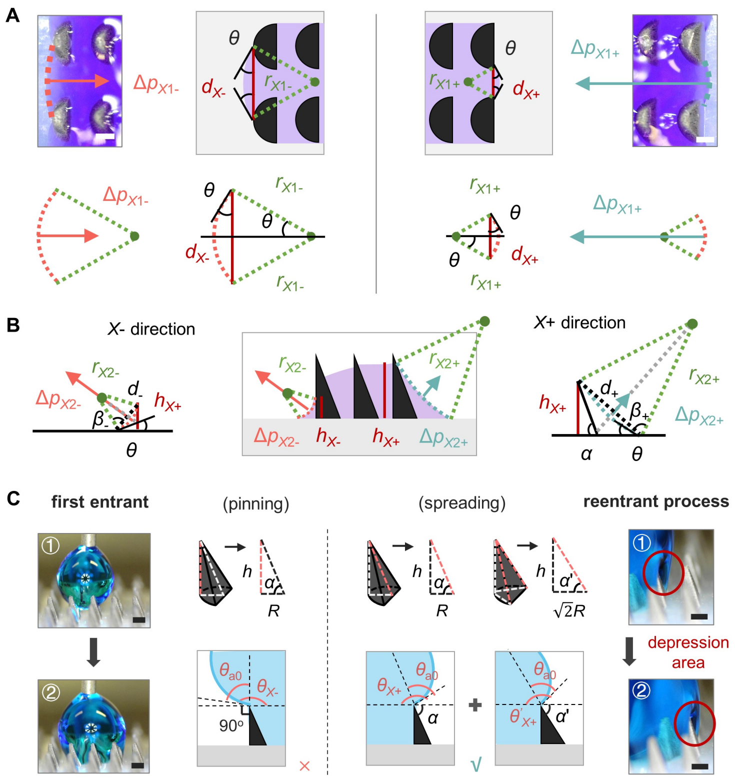

FIG. S11. Mechanism details of the two main liquid spreading modes. (A) Geometric relation for calculating $\Delta p_{X1\text{-}}$ and Δp_{X1+}. (B) Geometric relation for calculating $\Delta p_{X2\text{-}}$ and Δp_{X2+}. (C) Detailed mechanism of the reentrant mode. Scale bars: 500 µm.

VII. MULTIMODAL SPREADING MODES AND THE GENERALITY EVALUATION

In Figure 3D of the main text, we observe the multimodal directional liquid spreading behaviors that exhibit differences along the X, Y, and Z axes. Here we illustrate the detailed liquid spreading process for liquids belonging to six different ethanol-to-water ratios ($\chi = 100\%, 80\%, 60\%, 40\%, 15\%,$ and 0%), and explain the underlying mechanism for the transition in spreading behaviors as the surface tension of the liquid increases. For the straight-through mode, the pinning effect on the liquid along the X- direction, Y-axis, and $X+$ direction is successively strengthened when χ decreases. Consequently, as the liquid spreading is significantly influenced by this increased pinning, its spreading in these directions becomes progressively restricted (mode ①–③, Figure S12A-C). When χ continuously decreases, the liquid begins to climb over the top of the microratchet and eventually spreads along the Y-axis again (mode ④, Figure S12D). At approximately $\chi = 15\%$, the two main spreading modes achieve a balance, resulting in bidirectional liquid spreading (mode ⑤, Figure S12E). For liquids with higher surface tension ($\chi = 0\%$), the liquid does not tend to wet the rectifier surface, and the bottom pinning becomes exceptionally strong. Therefore, these liquids exhibit a standard reentrant mode (mode ⑥, Figure S12F).

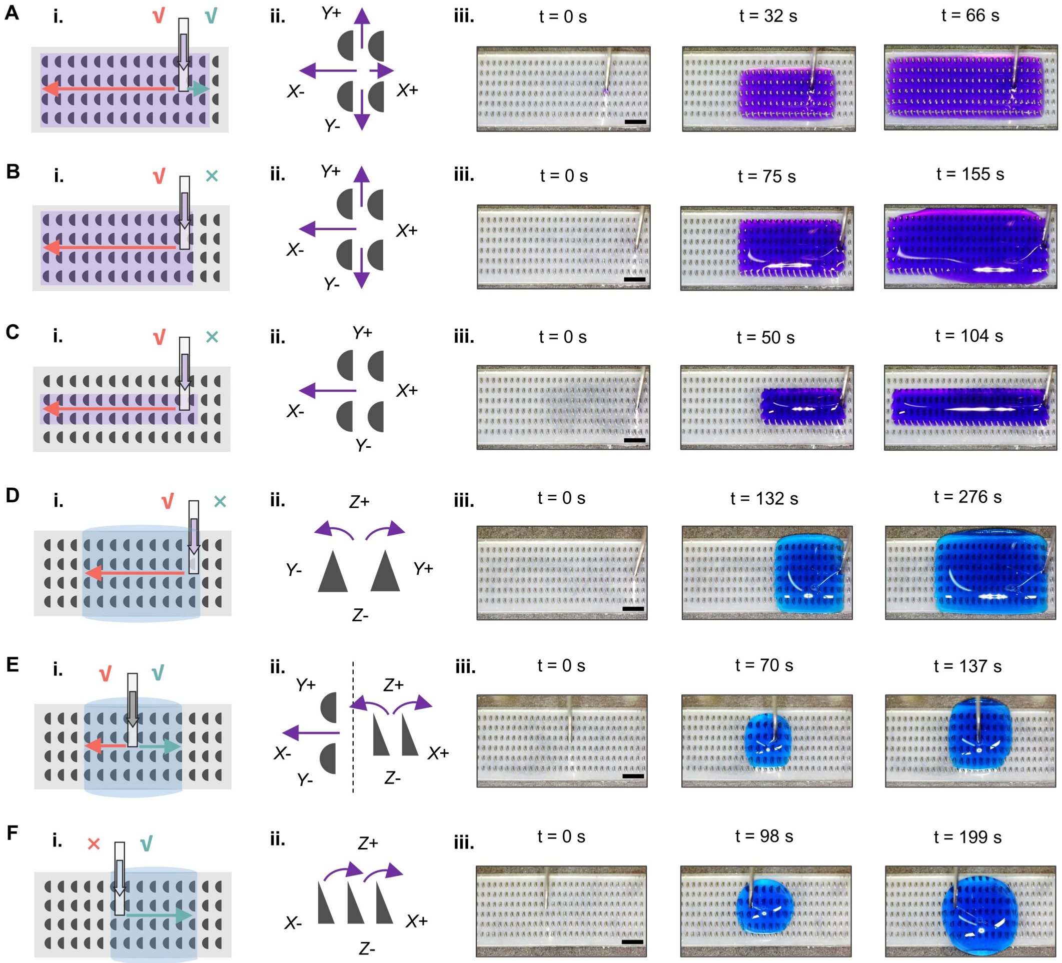

FIG. S12. Multimodal liquid spreading and their transition. (A)-(F) Spreading modes ①–⑥: (i)-(ii) schematics and (iii) experiments. Scale bars: 3 mm.

Figure 3Diii of the main text shows the change in liquid spreading area over time, which indicates the variation in liquid spreading velocity. Essentially, liquid spreading velocity is correlated with two primary influencing factors: the liquid injection flow rate and the liquid surface tension (Figure S13A). In our liquid-infused model, a higher injection flow rate compels the liquid to direct and spread more rapidly, thereby triggering a faster liquid spreading velocity. In addition, high surface tension liquids exhibit greater accumulation height on the rectifier, resulting in a lower liquid spreading velocity at the same flow rate. We utilize the change in liquid spreading area over time to quantify the liquid spreading velocity in the XY-plane. As shown in Figure S13B, the liquid spreading velocity decreases with the decrease of χ, while a high liquid injection flow rate (300 µL/min) leads to a greater spreading velocity for the same surface tension liquids than the low injection flow rate (100 µL/min).

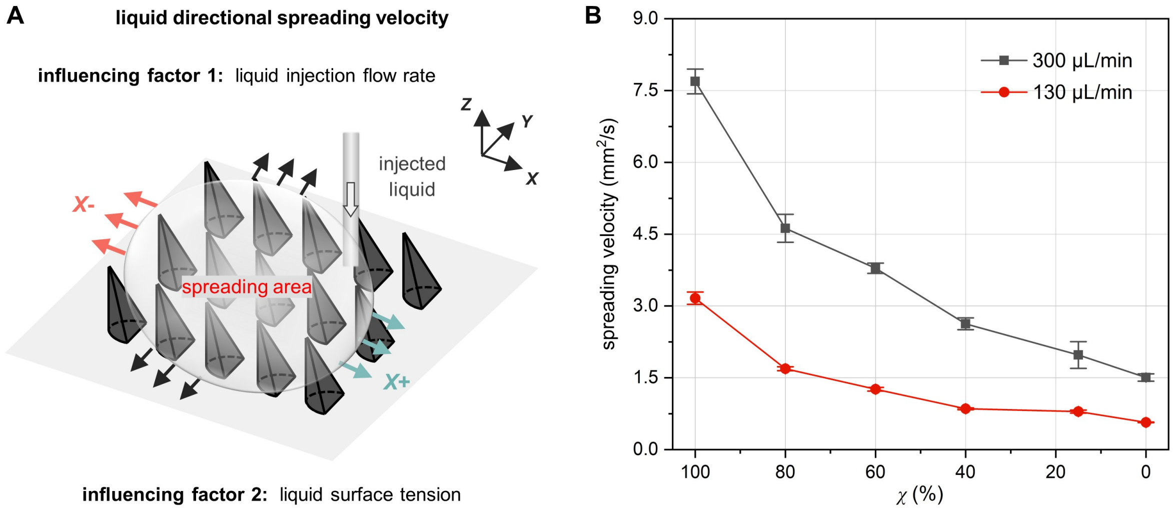

FIG. S13. Evaluation on the liquid spreading velocity. (A) Two main influencing factors: liquid injection flow rate and liquid surface tension. (B) The spreading velocity of liquids with different surface tensions at injection flow rates of 130 µL/min and 300 µL/min.

The evaluation of the influence of inertial effects on the liquid spreading mode is based on the We-χ phase diagram (Figure 3E-F of the main text). We demonstrate how the two main modes display distinct characteristics influenced by inertia via changing the scale of the system. When a specific size parameter undergoes a slight change (<±50%), the fundamental features of these We-χ phase diagrams remain qualitatively similar, despite small shifts observed in the critical value and mode transition value. As in Figure S14A, the increased horizontal spacing d_{h}, as a buffer space, raises the critical value of liquids following the straight-through mode. It allows the liquid to withstand higher injection flow rates while maintaining the original directional spreading mode. In contrast, the increased vertical spacing d_{v} weakens the Laplace pressure, thereby facilitating an earlier transition from the straight-through mode to bidirectional spreading (Figure S14B). When the height of the microratchet h is reduced, it becomes easier for the liquid to climb over the microratchet in the reentrant mode. This results in a higher ethanol concentration for the transition between the two primary modes to occur (Figure S14C).

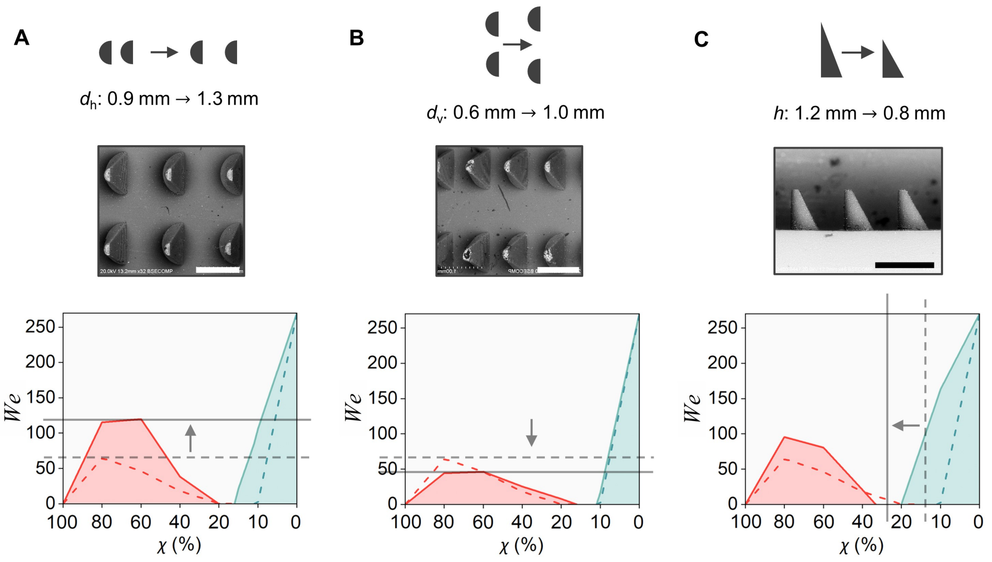

FIG. S14. We-χ phase diagrams under varying size parameters. (A) Increasing d_{h} from 0.9 mm to 1.3 mm will increase the critical liquid injection speed for the straight-through mode. (B) Increasing d_{v} from 0.6 mm to 1.0 mm will decrease the critical liquid injection speed for the straight-through mode. (C) Decreasing h from 1.2 mm to 0.8 mm makes the critical ethanol concentration for mode transition larger. Scale bars: 1 mm.

We also evaluate the scale effect on the rectifier's multimodality in liquid spreading (Figure 3G of the main text). We introduce a scale coefficient to describe the magnification/reduction of the rectifier with respect to the original design used in the main text. This change in scale maintains the proportion of the original size parameters. For example, when the scale coefficient is equal to 0.2, it means that the original design size parameters, including R = 500 µm, h = 1200 µm, d_h = 900 µm, and d_v = 600 µm, are changed to R = 100 µm, h = 240 µm, d_h = 180 µm, and d_v = 120 µm. The rectifiers corresponding to different scale coefficients mentioned in the main text are depicted in Figure S15. When the scale of the rectifier is reduced, its multimodal rectification effect can still be maintained. However, if the rectifier size becomes too large, the multimodal behavior will be lost, and all the liquids (23–72 mN/m) will predominantly follow the straight-through spreading mode (Figure 3G of the main text).

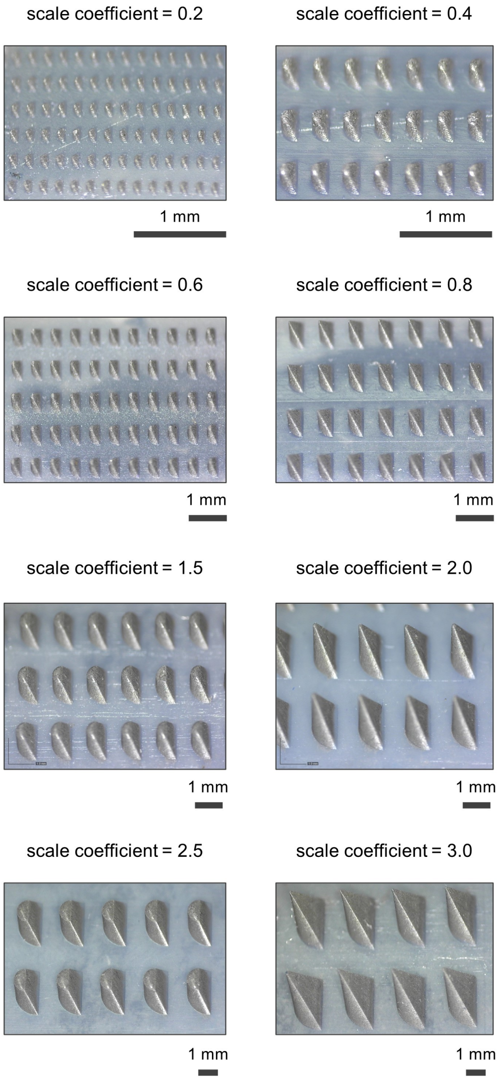

FIG. S15. Illustration of the rectifiers with different scale coefficients.

It is important to note that the transition between the two main modes cannot be solely determined by whether the liquid can cross over the top of microratchets. As illustrated in Figure S16A, when utilizing a rectifier with a scale coefficient of 0.2, even pure ethanol can immerse the top of microratchets. However, the spreading is still influenced by the asymmetric Laplace pressure, leading to a straight-through mode. Only when the Laplace pressure pins the liquid strong enough (occurring in high surface tension liquids), the low wettability liquid will show the reentrant spreading mode after exceeding the top of microratchets. Even in the case of high surface tension liquids, the liquid may not accumulate beyond the top of the ratchet (e.g., on the rectifier with a scale coefficient of 3, Figure S16B). At this time, the liquid will also exhibit the straight-through spreading mode.

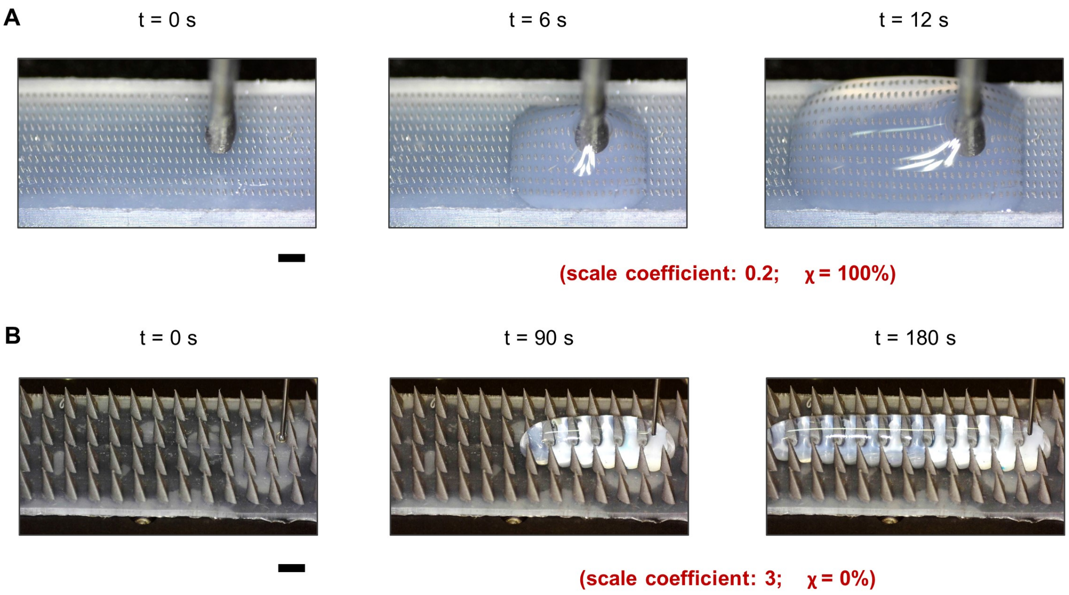

FIG. S16. Liquid rectification experimental results of rectifiers with different scale coefficients. (A) The liquid with $\chi = 100\%$ exhibits straight-through mode on a rectifier with a scale coefficient of 0.2. (B) The liquid with $\chi = 0\%$ exhibits straight-through mode on a rectifier with a scale coefficient of 3. Scale bars: 700 µm (A), and 5 mm (B).

VIII. REENTRANT SPREADING MODE UNDER THE RECONFIGURED RECTIFIERS

Figure S17 shows the impact of reconfigured microratchets on the reentrant mode. Reconfigured microratchets change the actual advancing contact angles of the liquid along the X- and $X+$ directions to adjust the liquid transport directions. The X- bending microratchet amplifies the existing top structural heterogeneity, leading to increased $\theta_{X\text{-}}$ and decreased θ_{X+}. As a result, the liquid follows the contact angle hysteresis principle and still spreads in the $X+$ direction. In contrast, the $X+$ bending microratchet alters the original top structural heterogeneity and results in $\theta_{X\text{-}} < \theta_{X+}$. Therefore, it changes the liquid's original $X+$ spreading to X- spreading.

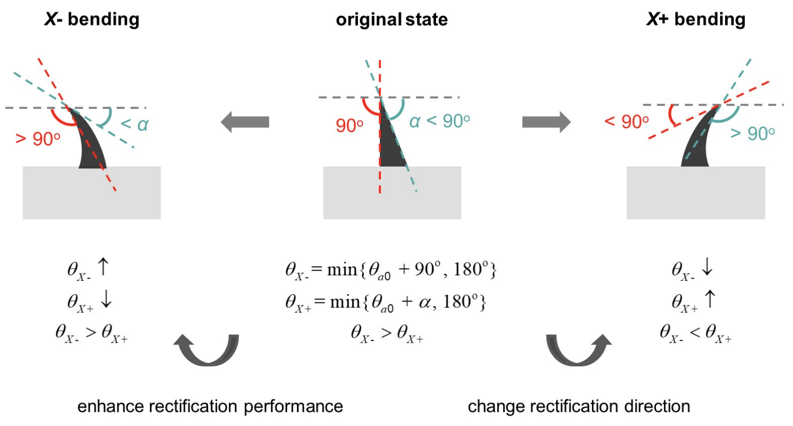

FIG. S17. Comparison of $\theta_{X\text{-}}$ and θ_{X+} in X- bending, $X+$ bending, and original state microratchets.

Under gradient magnetic fields, the reconfigured rectifier may pin the liquid spreading in both the X- and $X+$ directions, when injecting liquids at point B (Figure 4Fiii-iv of the main text). But unexpectedly, the liquid will finally present directional spreading towards the $X+$ direction. Such a new spreading behavior is attributed to the presence of a heterogeneous cross-section of the microratchet (Figure S18). In the X- direction, the flat surface blocks the liquid propulsion line and strongly pins the liquid (Figure S18A); while in the $X+$ direction, the curvature surface still allows the liquid to spread further (Figure S18B). Finally, the liquid will present the $X+$ directional spreading.

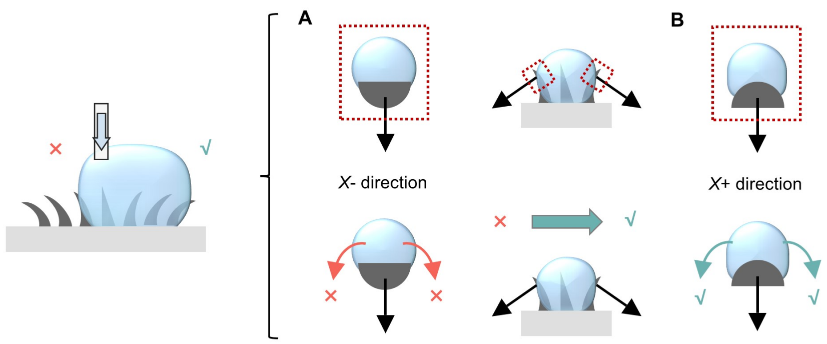

FIG. S18. Reconfigured microratchet (under gradient magnetic fields) mediated reentrant directional liquid rectification. (A) The liquid is pinned in the X- direction. (B) The liquid still can spread the $X+$ direction.

IX. CAPILLARITY-MEDIATED LIQUID PURITY TESTING

In capillarity-mediated liquid purity testing experiments (Figure 5A of the main text), the size of the rectifier is appropriately reduced, thus its narrow capillary channel width (w_c) provides a more intuitive capillary effect. The specific size parameters are shown in Figure S19A-B. To illustrate the thresholding effect of our design, the conventional model (straight wall) maintains equivalent w_c and h to our rectifiers. In addition, it is worth noting that the applied magnetic field promotes higher capillary height (h_c). Similar to promoting the straight-through mode (X- bending configuration in Figure 4A of the main text), the magnetic-field-driven microratchet bending leads to a larger net Laplace pressure to pull the liquid upward. Therefore, the recognition interval of the rectifier exhibits a wider range of h_c, like a detection amplifier (Figure S19C). In this novel capillary phenomenon, the continuity of the curvature microratchet along the Z-axis (i.e., the arrangement density) will influence the rising height of the liquid. However, when keeping the standard semicircular design, we expect the critical contact angle threshold that distinguishes $h_c > 0$ and $h_c = 0$ remains unchanged ($\sim 38^o$, i.e., the contact angle of $\chi = 90\%$ liquid on the rectifier). Considering the difference of detected liquids, it is necessary to judiciously select rectifier materials that can tailor the target detection liquid to exhibit near the critical contact angle on the rectifier. For instance, if the objective is to screen for high-purity ethanol (e.g., 95% or greater), more hydrophobic materials should be chosen to fabricate the rectifier; conversely, if the required screening purity can be relaxed to 75%, more hydrophilic materials should be chosen to fabricate the rectifier.

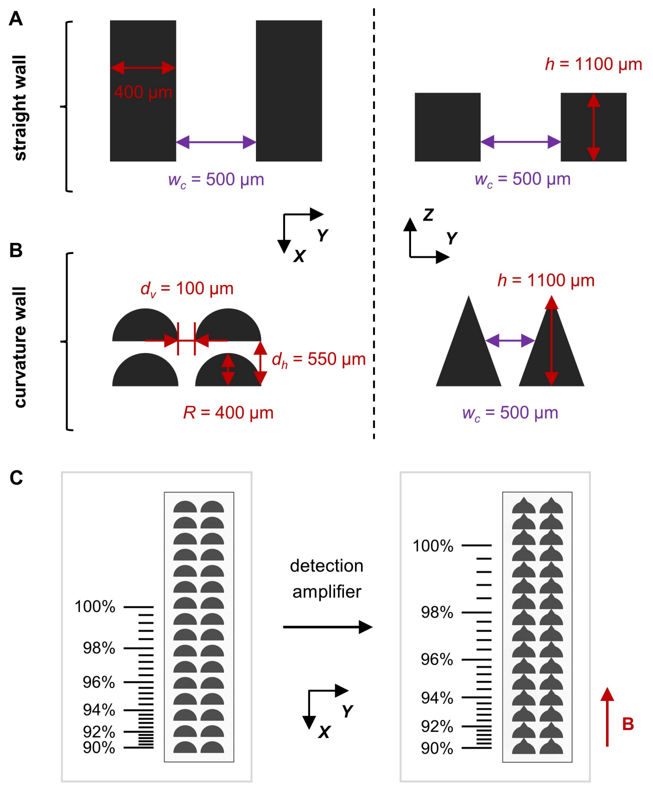

FIG. S19. (A) The design size parameters of the straight wall. (B) The design size parameters of the curvature wall. (C) Magnetically driven reconfigured rectifier with amplified capillarity height (h_c) range.

X. 3D LIQUID CRAWLER WITH ADJUSTABLE MORPHOLOGICAL PARAMETERS

In the main text, we illustrate the inchworm-like 3D liquid crawler and its morphological parameter definitions (Figure 5B). These parameters can be controllably adjusted by employing various liquids and strategically configuring magnetic fields (Figure S20Ai-iv). In general, this kind of liquid crawler can be formed and tailored in a specific liquid range, which is χ = 100%–70% in our rectifier design. When the applied magnetic field intensity does not change much, the crawler's initial height (h_0) and critical height (h_{arch}) are mainly decided by the liquid's surface tension. The initial length (l_0) of the crawler is determined by the width of the magnet below the injection position (w_{m0}); while the crawling step length (l_{step}) is influenced by the width of the neighboring magnet (w_{m1}). Based on the 3D liquid crawler in the main text (Figure S20Bi), we show how to regulate different morphological parameters by changing liquids or magnetic fields (Figure S20Bii-iv). When a liquid (χ = 90%) with a slightly higher surface tension is considered (Figure S20Bii), the liquid crawler exhibits a reduction in both h_0 and h_{arch} (Figure S20Ci). By utilizing type-1 magnet arrays (Figure S20Biii), the liquid crawler presents a decrease in both l_0 and l_{step} (Figure S20Cii-iii). Additionally, by combining type-1 and type-2 magnets (Figure S20Biv), the crawling step length (l_{step}) of the liquid crawler can be independently regulated (Figure S20Ciii).

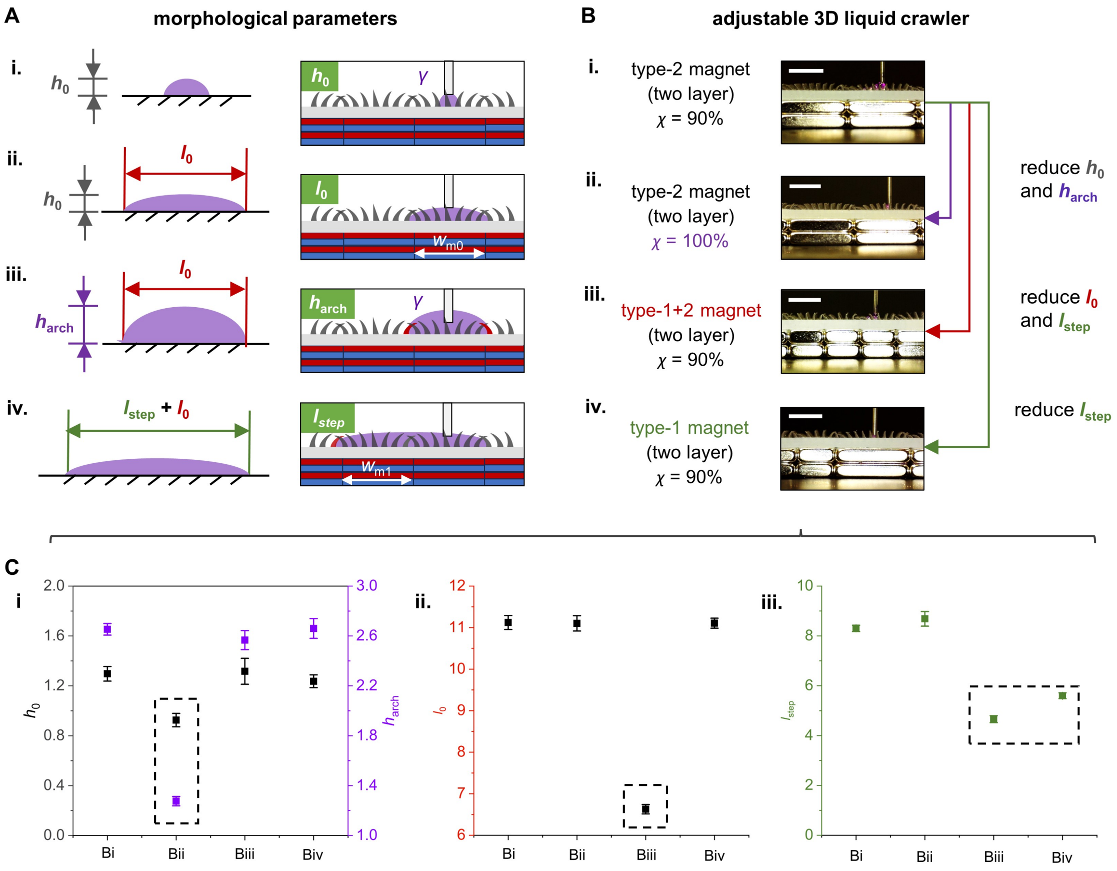

FIG. S20. (A) Morphological parameter definitions and their determined factors, including (i) initial height (h_0); (ii) initial length (l_0); (iii) critical height (h_{arch}); and (iv) crawling step length (l_{step}). (B) (i)-(iv) The arrangement of four types of liquid crawlers. (C) (i)-(iii) Adjusted morphological parameters in different liquid crawlers (mean ± SD). Scale bars: 5 mm (B).

XI. LOGICAL LIQUID TRANSPORT IN MULTI-CHANNELS

In this section, we illustrate the arrangement of rectifier and magnet arrays in logical liquid transport experiments (Figure 5C-E of the main text). Figure S21Ai shows the microratchet distribution of the four-channel liquid rectifier. The liquid following the straight-through mode preferentially spreads towards the X- and $Y+$ direction (Figure S21Aii) as shown in Figure 5D of the main text. By implementing gradient magnetic fields (two-layer type-3 magnets) beneath channels II and IV, we can reconfigure the rectifier (Figure S21Bi). This configuration enables the logical filling of liquid in the four channels in a sequential manner, enhancing the control and precision of the system (Figure S21Bii). Specifically, the gradient magnetic field causes the strongly curved ratchet to inhibit the spreading of the liquid. As a result, channels I and III will be filled preferentially compared to channels II and IV. Furthermore, according to the principles of straight-through liquid spreading, channel I will have priority over channel III in terms of liquid filling. Based on the rules of inchworm-like liquid spreading, channel II will take precedence over channel IV. These rules dictate the sequence in which the liquid will propagate through the channels, ensuring controlled and orderly filling based on the specific spreading mechanisms employed in each channel.

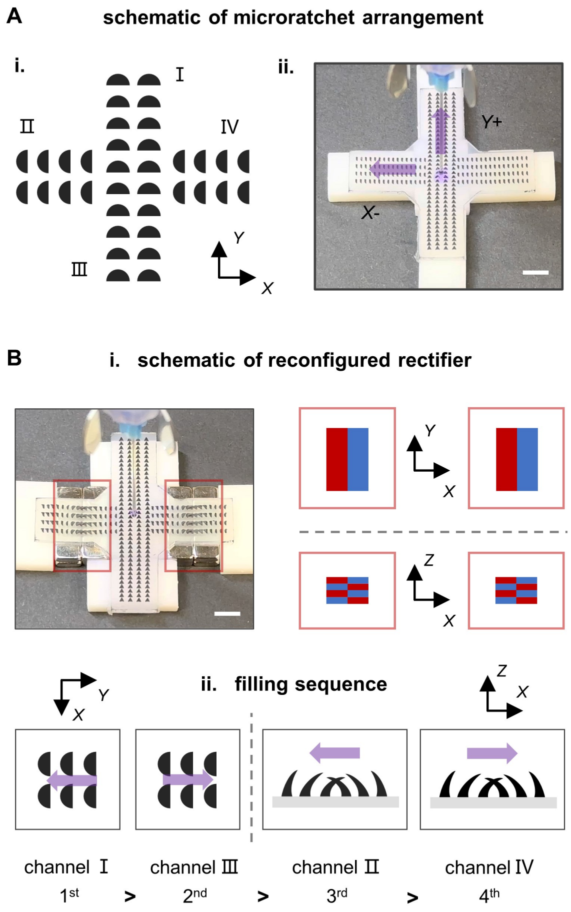

FIG. S21. (A) Schematic of the microratchet arrangement in the original rectifier configuration: (i) illustration; and (ii) experimental snapshot. (B) (i) Schematic of the reconfigured rectifier and its corresponding magnet array arrangement. (ii) The liquid's filling sequence in four channels. Scale bars: 5 mm.

XII. SUMMARY OF POTENTIAL APPLICATIONS

In this section, we briefly discuss the potential applications of our liquid manipulation paradigm. Benefiting from the novel binary capillary phenomenon, we envision the rectifier's potential value in rapid liquid identification. Based on the calibrated purity test criterion, we can visually separate qualified/unqualified liquids (Figure S22A). This distinction criterion is essentially based on liquid surface tension, therefore it is extremely suitable for testing organic solutions whose surface tension varies greatly with purity. Based on the calibrated capillary height, we can even determine its approximate concentration to a certain extent (e.g., ∼98%). The method is attractive because it does not require any electronic or optical equipment. Simply inserting a small rectifier in the liquid allows easy visualization of purity and rapid screening of large sample sets to identify qualified and unqualified liquid groups. Moreover, as illustrated in Figure 5C-E of the main text, the demonstrated logical liquid transport in multi-channels may show potential in complex open-channel microfluidics. Figure S22B presents the concept of using logical transport to achieve rich chemical reactions and biochemical analysis. By designing the microratchet arrangement and selectively setting the blocking magnetic field under each path, we can construct rich forms of liquid detection. The binary 0/1 logic based on the structured surfaces has limitations for complex liquid operations (Figure 5D of the main text), but incorporating magnetic fields creates diverse logical relationships to improve this (Figure 5E of the main text). Importantly, magnetic fields can be implemented using small magnets, enabling a portable and cost-effective solution. Given that the magnetic field is pre-customized, our liquid manipulation paradigm could allow non-expert operators to easily implement pre-set liquid operations, thus potentially serving as point-of-care devices. A conceptual diagram of the device is shown in Figure S22C, which would be portable, wireless, and cost-effective. Customizing the rectifier and underlying magnetic field for different products could make it more practical and widely applicable.

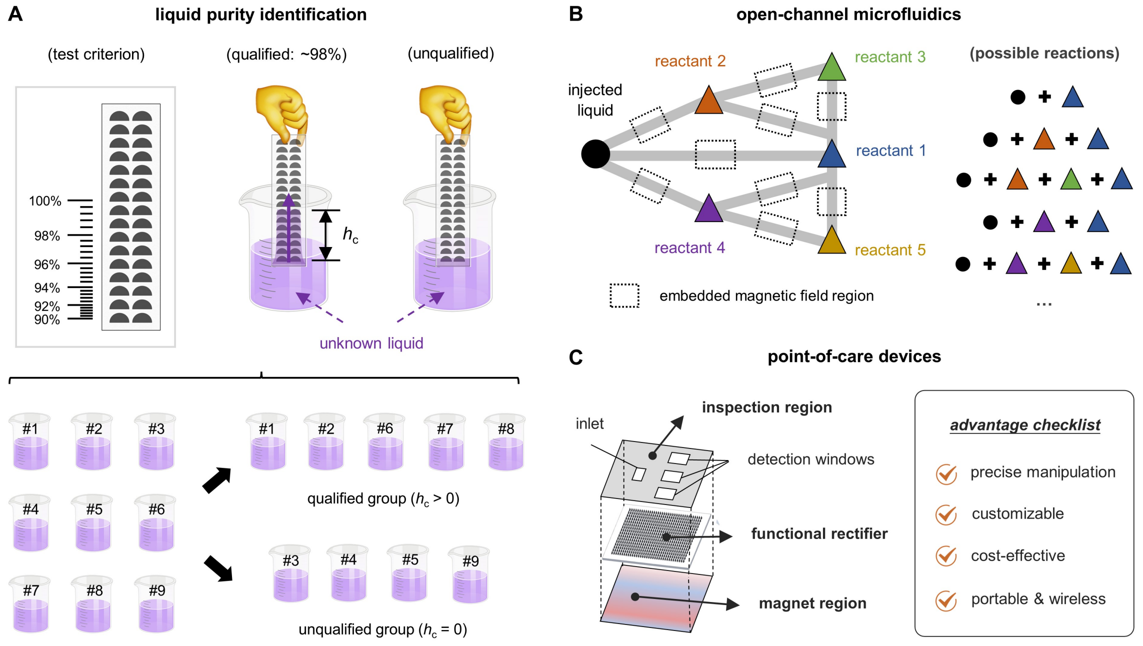

FIG. S22. Potential applications of the proposed liquid manipulation paradigm in (A) liquid purity identification; (B) open-channel microfluidics; (C) point-of-care devices.

Legends for Movies S1-S5

Movie S1. Multimodal 3D liquid spreading behaviors via the hierarchical rectifier.

Movie S2. Reconfigured rectifiers with enhanced flexibility in 3D liquid manipulation.

Movie S3. Inchworm-like 3D liquid spreading.

Movie S4. Logical liquid transport in multiple open channels.

Movie S5. Spatiotemporally controlled chemical reaction platforms.

[1] D. Gross, W. Hauger, J. Schröder, W. A. Wall, and J. Bonet, *Engineering mechanics 2* (Springer, 2011).
